# Quality of family planning services in HIV integrated and non-integrated health facilities in Malawi and Tanzania

**DOI:** 10.1186/s12978-019-0712-y

**Published:** 2019-05-29

**Authors:** Michael A. Close, Janine Barden-O’Fallon, Carolina Mejia

**Affiliations:** 1 Carolina Population Center, Health Behavior Department, Gillings School of Global Public Health, University of North Carolina at Chapel Hill, Chapel Hill, NC USA; 2 Carolina Population Center, Maternal & Child Health Department, Gillings School of Global Public Health, University of North Carolina at Chapel Hill, Chapel Hill, NC USA; 3 Carolina Population Center, University of North Carolina at Chapel Hill, Chapel Hill, NC USA

**Keywords:** Service quality, Quality index, Integration, Family planning, HIV, SPA, QIQ, Malawi, Tanzania

## Abstract

**Background:**

The integration of family planning (FP) and HIV-related services is common in sub-Saharan Africa. Little research has examined how FP quality of care differs between integrated and non-integrated facilities. Using nationally representative data from Malawi and Tanzania, we examined how HIV integration was associated with FP quality of care.

**Methods:**

Data were drawn from Service Provision Assessments (SPAs) from Malawi (2013–2014) and Tanzania (2014–2015). The analytic sample was restricted to lower-level facilities in Malawi (*n* = 305) and Tanzania (*n* = 750) that offered FP services. We matched SPA measures to FP quality of care indicators in the Quick Investigation of Quality (QIQ). We conducted bivariate and multivariate analyses of 22 QIQ indicators to examine how integration status was related to individual QIQ indicators and overall FP quality of care at the facility- and client-level.

**Results:**

The prevalence of HIV integration in Malawi (39%) and Tanzania (38%) was similar. Integration of HIV services was significantly associated (*p* < 0.05) with QIQ indicators in Malawi (*n* = 3) and Tanzania (*n* = 4). Except for one negative association in Tanzania, all other associations were positive. At the facility-level, HIV integration was associated with increased odds of being at or above the median in FP quality of care in Malawi (adjusted odd ratio (OR) = 2.24; 95% confidence interval (CI) = 1.32, 3.79) and Tanzania (adjusted OR = 2.10; 95% CI = 1.37, 3.22). At the client-level, HIV integration was not associated with FP quality of care in either country.

**Conclusion:**

Based on samples in Malawi and Tanzania, HIV integration appears to be beneficially associated with FP quality of care. Using a spectrum of FP quality of care indicators, we found little evidence to support concerns that HIV integration may strain facilities and providers, and adversely impact quality outcomes. Rather, it appears to strengthen FP service delivery by increasing the likelihood of stocked FP commodities and achievement of other facility-level quality indicators, potentially through HIV-related supply chains. Further research is needed to assess FP quality of care outcomes across the various platforms of FP integration found in sub-Saharan Africa.

**Electronic supplementary material:**

The online version of this article (10.1186/s12978-019-0712-y) contains supplementary material, which is available to authorized users.

## Background

Integration of family planning (FP) and HIV-related services is a long-term trend in the health systems of sub-Saharan Africa [1]. While FP has been integrated with myriad other health services (e.g. maternal, neonatal, and child health services) [2], HIV-related services are a foremost platform of integration due to the prevalence of HIV and AIDS in the region [1]. We define service integration as the delivery of two different types of health services at the same facility, though various more precise definitions of integration are present in the literature [1, 3]. Integrated service delivery has grown more prevalent as research has accumulated on its potentially favorable effects, and as it has gained the support of local stakeholders [4].

The literature on FP and HIV service integration effects on facility-, provider-, and client-level outcomes is largely positive though inconclusive. Integration of FP and HIV services has been associated with beneficial clinical outcomes (e.g. prevention of unintended HIV-positive births) [5, 6], service delivery outcomes (e.g. improved service uptake) [7], and cost effectiveness (e.g. cost savings from infant HIV infections prevented) [1, 8]. However, the infrastructural and logistical challenges of integrating health services [4] has the potential to negatively affect base service quality [9] and dilute providers’ expertise [10].

Despite the predominance of integrated FP and HIV services, there is a paucity of research on how integrated programming may impact FP quality of care. The maintenance of FP quality of care is essential for positive client health outcomes and adherence to a reproductive rights-based approach to FP. The Bruce/Jain Quality of Care Framework, which has guided the design and delivery of services in the field of FP for over two decades, outlines six critical elements that constitute FP quality of care: choice of methods, information given to users, technical competence, interpersonal relations, follow-up or continuity mechanisms, and appropriate constellation of services [11]. The multidimensional nature of FP quality of care posited by the Bruce/Jain Framework necessitates measurement at the facility level (e.g. availability of FP methods), provider level (e.g. adherence to infection control guidelines), and client level (e.g. communication about client-preferred FP method) to sufficiently capture FP quality of care.

The few studies available on FP quality of care in HIV integrated facilities are limited by methodological concerns. In a review of integrated FP and HIV services globally, Spaulding and colleagues [12] identified four studies [13–16] reporting on “quality of services,” though most of this research is either drawn from grey literature with insufficiently detailed or weak study design [14–16] or reliant on provider-reported information such as knowledge and attitudes [16] as a proxy for FP quality of care. To gain a comprehensive understanding of FP quality of care, a theory-based measurement approach comprising indicators of quality at the facility, provider, and client level is needed for evidence-informed decision making.

The present study aimed to fill these knowledge gaps by using multiple objective indicators from a theory-based FP quality of care measurement tool, Quick Investigation of Quality (QIQ), to assess the quality of FP care among HIV integrated and non-integrated facilities using Service Provision Assessment (SPA) data from Malawi (2013–2014) and Tanzania (2014–2015). Our specific objectives for this study were to investigate the level of FP quality of care in HIV integrated and non-integrated facilities, how FP quality of care compares between HIV integrated and non-integrated facilities, and finally, to determine the degree to which integration is associated with FP quality of care when controlling for other facility characteristics. We hypothesized that there are differences in the quality of FP service provision between HIV integrated vs. non-integrated facilities.

## Methods

### Study design and data sources

We conducted a retrospective cross-sectional study based on secondary datasets from recent SPA conducted in Malawi (2013–2014) and Tanzania (2014–2015). The purpose of the SPA is to assess the availability and quality of basic and essential health services to identify gaps and compare findings across health systems [17, 18]. Four types of data collection instruments are used to understand relevant facility-, provider-, and client-level characteristics: Facility Inventory Questionnaire, Health Provider Interview Questionnaire, Observation Protocols for selected health services (including FP), and Exit Interview Questionnaires for selected clients and caretakers (including FP clients). In sum, these data collection tools provide a comprehensive snapshot of the status of a wide range of basic and essential health services, including those related to FP and HIV.

We used data collected from the Facility Inventory Questionnaire, FP Observation Protocol, and FP Client Exit Interview Questionnaire. The data collection methodology for Malawi and Tanzania were largely identical [17, 18]. A team of data collectors visited each facility to administer questionnaires and observation protocols. For the Facility Inventory Questionnaire, a data collector approached knowledgeable staff members with relevant information to complete each section. For the FP Observation Protocol, data collectors were instructed to observe a maximum of five clients for each provider of the service, with a maximum of 15 observations per service per facility. If several eligible FP clients were present and waiting for an appointment, interviewers sought to select two new clients for every follow-up client. Each client with an observed consultation was approached afterward to complete the FP Client Exit Interview Questionnaire. If the service was not offered on the day that data collectors arrived, there would be a return visit to administer the relevant observation protocol and interviews. However, no return visit was conducted if the service was offered on that day, but no clients came for the service. Consequently, not all facilities in the sample have FP Observation Protocol and FP Client Exit Interview data. Further details on the SPA are reported elsewhere [17–19].

### Sample

**Fig. 1:**
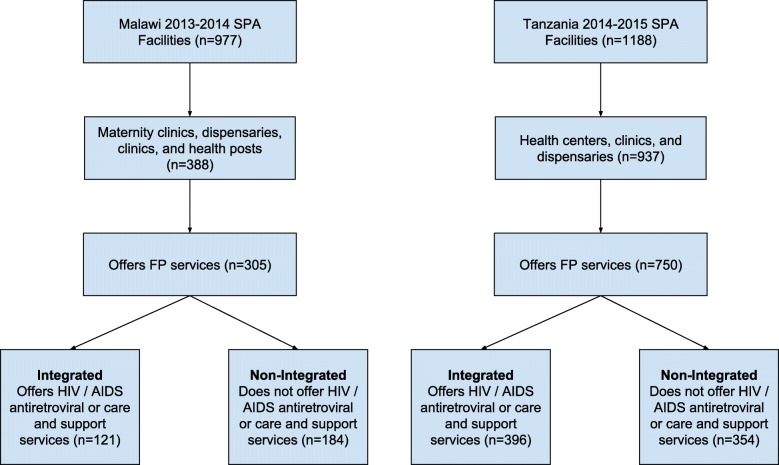
Study Inclusion of Facilities from Malawi SPA 2013–2014 and Tanzania SPA 2014–2015 Data

Facilities included in the sample offered any FP services, as recorded in the general service availability section of the facility inventory. In the present study, we define HIV integration as a facility that offers FP services in addition to offering either “HIV/AIDS antiretroviral prescription or antiretroviral treatment follow-up services” or “HIV/AIDS care and support services, including treatment of opportunistic infections and provision of palliative care.” Facilities were considered “non-integrated” if they offered HIV testing and counseling services but neither of the two categories of HIV care and support services. (Note that HIV testing and counseling is a common practice; of facilities offering FP services, 85% in Malawi and 98% in Tanzania also offered HIV testing and counseling services and nearly all facilities included in the analysis in Malawi (119/121) and Tanzania (394/396) provided at least one long acting reversible contraceptive (LARC) method.) Importantly, both integrated and non-integrated facilities offered a variety of primary health care services, such as, antenatal care and child health services, in addition to FP. Figure 1 displays a study flowchart of the sample strategy.

### Malawi

The Malawi SPA was a census of all formal-sector facilities in the country. In Malawi, 977 of 1060 (92%) facilities were assessed and included in the SPA dataset. Facilities in the sample frame not assessed were for reasons of: refusal (3%), closed down/not yet operational (2%), no respondent available (1%), and inaccessibility (2%). Stratification by type of facility showed that, of the 505 hospitals and health centers offering FP services, only 20 (4%) were non-integrated. Since integration is almost 100% at these types of facilities, they were excluded from the analysis, leaving 388 maternity clinics, dispensaries, clinics, and health posts. Of the 388 facilities, 305 (79%) offer FP services. Of facilities offering FP services, 121 (40%) also offered “HIV/AIDS antiretroviral prescription or antiretroviral treatment follow-up services” or “HIV/AIDS care and support services, including treatment of opportunistic infections and provision of palliative care.” Therefore, the facility-level analytic sample (*n* = 305) had a smaller proportion of facilities that were HIV integrated (40%) as compared to non-integrated (60%). Of the 305 facilities, 108 facilities had FP client observations (*n* = 323) and FP client exit interviews (*n* = 315), and constitute the client-level analytic sample.

### Tanzania

The Tanzania SPA was a nationally representative probability-based sample survey of all formal-sector facilities in the country. In Tanzania, 1188 of 1200 facilities (99%) sampled were assessed and included in the SPA dataset. Facilities that were sampled but not assessed (1%) were due to refusal (*n* = 7), closed down/not yet functional (*n* = 4), and inaccessibility (*n* = 1). Stratification by type of facility showed that, of the 183 hospitals offering FP services, only eight (4%) were not HIV integrated. As in Malawi, integration was almost 100% at this level of service provision. Excluding hospitals from the analysis resulted in 937 health centers, clinics, and dispensaries. Of these, 750 (80%) offer FP services. Of facilities offering FP services, 396 (53%) also offered “HIV/AIDS antiretroviral prescription or antiretroviral treatment follow-up services” or “HIV/AIDS care and support services, including treatment of opportunistic infections and provision of palliative care.” Therefore, the facility-level analytic sample (*n* = 750) was roughly evenly divided by integrated (53%) and non-integrated (47%) status. Of the 750 facilities, 365 facilities had FP client observations (*n* = 1060) and FP client exit interviews (*n* = 1059), and comprise the client-level analytic sample.

### Measures

#### Indicators

Indicators from the QIQ were mapped onto SPA measures to create indicators of FP quality of care (Table 1) [20]. The QIQ was developed by the MEASURE Evaluation Project to provide a rapid and low-cost methodology that can be used to routinely measure quality of care in clinic-based family planning programs and related reproductive health services. The QIQ comprises 25 indicators that measure five of six elements from the Bruce/Jain Framework of Quality of Care: choice of methods, information, technical competence, interpersonal relations, and follow-up [11, 20]. The data collection methodology was similar to the SPA in that a Facility Audit Questionnaire, FP Observation Protocol, and Client Exit Interview were developed to assess multiple levels of FP quality of care.

**Table 1:** List of Indicators in the QIQ

QIQ Indicator number	QIQ Indicator Description
1	Provider demonstrates good counseling skills^a^
1a	Look and write on client record
1b	Used any visual aids
1c	Ensured visual and auditory privacy
2	Provider assures client of confidentiality
3	Provider asks client about reproductive intentions
4	Provider discusses with client which method she would prefer
5	Provider mentions HIV/AIDS (initiates or responds)
6	Provider discusses dual method use
7	Provider treats client with respect/dignity^b^
8	Provider tailors key information to the particular needs of the specific client
9	Provider gives accurate information on the method accepted (e.g. how to use, side effects, and complications)
10	Provider gives instruction on when to return
11	Provider follows infection control procedures outlined in guidelines
12	Provider recognizes/identifies contraindications consistent with guidelines^b^
13	Provider performs clinical procedures according to guidelines
14	Staff treats client with dignity and respect
15	Client participates actively in discussion and selection of method (i.e. client is “empowered”)
16	Client receives her method of choice^b^
17	Client believes the provider will keep her information confidential^b^
18	Facility has all (approved) methods available; no stock-outs
19	Facility has basic items needed for delivery of methods available through facility (sterilizing equipment, gloves, blood pressure cuff, specula, adequate lighting, and water)
20	Facility offers privacy for pelvic exam/IUD insertion (no one can see)^c^
21	Facility has mechanisms to make programmatic changes based on client feedback
22	Facility has received a supervisory visit in past 6 months^d^
23	Facility has adequate storage of contraceptives and medicines (away from water, heat, and direct sunlight) on premises
24	Facility has state-of-the-art clinical guidelines
25	Waiting time is acceptable

^a^Treated as three sub-indicators in the analysis (1a-c)

^b^No match available

^c^Excluded

^d^We set the cut-off at 6 months

We matched SPA measures to 21 of 25 original QIQ indicators. However, we excluded one of those QIQ indicators (Indicator 20) due to very few clients in the Malawi and Tanzania analytic samples with information on that indicator. Because we treated Indicator 1 as three separate sub-indicators in the analyses, 22 QIQ indicators were used in our analyses. Each indicator was operationalized as a dichotomous variable. An additional file presents the SPA mapping and dichotomization of each QIQ indicator [see Additional file 1, Table S1].

### Data analysis

Bivariate analyses were conducted to compare each QIQ indicator by HIV integrated vs. non-integrated status. The Pearson’s Chi-square test was used to examine whether the distribution of each QIQ indicator significantly differed by integration status. Results are presented as percentages.

Logistic regression analyses were used to determine if integration status was associated with FP quality of care. We estimated unadjusted models of the relationship between integration status and FP quality of care at both the facility- and client-level. In addition, we estimated adjusted models at the facility-level controlling for managing authority (Malawi: 1 = government/public, 2 = private [non-profit], and 3 = private [for profit]; Tanzania: 1 = government/public, 0 = not government/public), facility type (Malawi: 1 = dispensary, 2 = clinic, 3 = health post/maternity; Tanzania: 1 = health center/clinic, 2 = dispensary), zone (for Malawi) or region (for Tanzania), and urban/rural location (1 = rural, 0 = urban). The FP quality of care dependent variables for the facility- and client-level models were sum scores of the respective facility- and client-level QIQ indicators dichotomized at the median. The sum scores included all facility- and client-level indicators except Indicator 9 (Provider gives accurate information on the method accepted [how to use, side effects, complications]) and Indicator 13 (Provider performs clinical procedures according to guidelines), because very few respondents received a relevant service offering, and thus, had no information on the indicator. The facility-level FP quality of care dependent variable included seven indicators, and the sum score ranged 0–7 for both Malawi (median = 4, standard deviation [SD] = 1.64) and Tanzania (median = 4, SD = 1.39). The client-level FP quality of care dependent variable (with indicators from observations and client exit interviews) had 13 indicators and the median score ranged 1–13 for both Malawi (median = 7, SD = 2.03) and Tanzania (median = 7, SD = 2.04).

Analyses were weighted for sample design (Tanzania) and non-response (Malawi) to compensate for any over- or under-representation of facility type in the data. In client-level models, we specified the facility as the primary sampling unit to adjust standard errors for the clustering of clients within facilities. A two-tailed alpha of 0.05 was set for statistical significance. All analyses were conducted in Stata 15.0 (College Station, TX, USA).

## Results

### Distribution of facilities by country

#### Malawi

Of 305 facilities included in the weighted Malawi SPA analytic sample, 39% met the criteria for HIV services integration by offering FP services and at least one of the two HIV care and support services. Table 2 displays facility characteristics in the Malawi sample by integration status. The managing authority of integrated and non-integrated facilities did not significantly differ, with a majority of the sample reporting a private (for profit) managing authority (61%). Most integrated and non-integrated facilities were clinics (80%), though a higher proportion of integrated facilities were a dispensary (18%) than non-integrated facilities (10%). While the location of a facility in an urban or rural setting did not significantly differ by integration status (*p* = 0.156), a significantly higher proportion (*p* = 0.006) of integrated facilities were concentrated in northern (17%) and southeastern (24%) zones than non-integrated facilities.

**Table 2:** Characteristics of non-integrated and integrated facilities (Malawi SPA 2013–2014) (n = 305)

	Non-Integrated (*n* = 184)		Integrated (*n* = 121)		Total (*n* = 305)		*p-value*
	n	%	n	%	n	%	
Managing Authority							0.155
Government /Public	32	18.1	33	27.4	65	21.8	
Private (non-profit)^a^	33	17.7	20	16.4	53	17.2	
Private (for profit)^b^	119	64.2	68	56.1	187	61.0	
Facility Type							0.016
Dispensary	20	10.4	21	17.5	41	13.2	
Clinic	148	79.9	97	80.0	245	80.0	
Health post/ Maternity^c^	16	9.7	3	2.5	19	6.8	
Location							0.156
Urban	90	48.6	69	56.9	159	51.9	
Rural	94	51.4	52	43.1	146	48.2	
Zone							0.006
Northern	18	9.2	21	16.6	39	12.1	
Central east	30	16.0	7	5.7	37	11.9	
Central west	48	25.7	36	29.4	84	27.2	
Southeast	29	16.1	28	23.9	57	19.2	
Southwest	59	33.0	29	24.4	88	29.7	

^a^Private (non-profit) is composed of facilities that reported “Christian Health Association of Malawi (CHAM)”, “Mission/Faith-Based (Other than CHAM)”, or “non-government organization”

^b^Private (for profit) is composed of facilities that reported a “Private (for profit)” or “Company” managing authority.

^c^Due to the small number of maternities in the analytic sample, “Maternity” and “Health post” were collapsed into a single combined facility type

#### Tanzania

Of 750 facilities included in the weighted Tanzania SPA analytic sample, 38% were HIV integrated, as defined by offering FP services and at least of the two HIV care and support services. Table 3 displays facility characteristics for Tanzanian facilities. Nearly all facilities in the sample reported a government/public managing authority (88%) and rural location (83%). Most integrated (76%) and non-integrated (95%) facilities in the sample were a dispensary. However, a significantly higher proportion of integrated facilities (*p* < 0.0001) were a health center or clinic (24%) than non-integrated facilities (5%).

**Table 3:** Characteristics of non-integrated and integrated facilities (Tanzania SPA 2014–2015) (*n* = 750)

	Non-Integrated (*n* = 354)		Integrated (*n* = 396)		Total (*n* = 750)		*p*-value
	n	%	n	%	n	%	
Managing Authority							0.260
Government/Public	284	86.4	360	90.3	644	87.9	
Not Government/Public^a^	70	13.6	36	9.7	106	12.1	
Facility Type							< 0.0001
Health center/Clinic^b^	82	5.0	263	24.1	345	12.2	
Dispensary	272	95.0	133	76.0	405	87.8	
Location^c^							0.543
Urban	70	15.7	80	18.0	150	16.6	
Rural	284	84.3	316	82.0	600	83.4	

^a^Government/Public is comprised of facilities that reported a “Private”, “Mission/Faith-Based”, or “Other (Parastatal and defense/prison/police)”

^b^Due to the small number of clinics in the analytic sample, “Clinic” and “Health center” were collapsed into a single combined facility type

^c^Region estimates not shown

### Distribution of facilities meeting QIQ indicators, by integration status: bivariate analyses

#### Malawi

Eleven of 22 QIQ indicators were reported as met by at least half of facilities and clients in each integration category of the Malawi analytic sample (Table 4). Of seven facility-level indicators, only three were met by at least half of facilities in each integrated and non-integrated category. Of fifteen client-level QIQ indicators, eight were reported as met by at least half of clients in each integrated and non-integrated facility category.

**Table 4:** Percentage of non-integrated and integrated facilities and clients meeting each QIQ Indicator (Malawi SPA 2013–2014)

Indicator#	Description	Non-Integrated	Integrated	Total	*p*-value
Facility-level Inventory (*n* = 305)		%	%	%	
11	Provider (at facility) follows infection control procedures outlined in guidelines	60	63	61	0.620
18	Facility has all (approved) methods available; no stockouts	46	58	50	0.039
19	Facility has basic items needed for delivery of methods available through facility (sterilizing equipment, gloves, blood pressure cuff, specula, adequate lighting, water)	36	35	36	0.902
21	Facility has mechanisms to make programmatic changes based on client feedback	25	30	27	0.370
22	Facility has received a supervisory visit in past 6 months^a^	64	80	71	0.003
23	Facility has adequate storage of contraceptives and medicines (away from water, heat, direct sunlight) on premises	69	78	72	0.087
24	Facility has state-of-the-art clinical guidelines	45	48	46	0.629
Client-level FP Observation (*n* = 323)					
1a	Look and write on client record	84	96	89	0.005
1b	Used any visual aids	20	21	20	0.907
1c	Ensured visual and auditory privacy	90	82	87	0.334
2	Provider assures client of confidentiality	32	22	28	0.266
3	Provider asks client about reproductive intentions	29	26	28	0.688
5	Provider mentions HIV/AIDS (initiates or responds)	8	15	11	0.207
6	Provider discusses dual method use	6	15	10	0.095
9	Provider gives accurate information on the method accepted (how to use, side effects, complications)^b^	57	63	60	0.552
10	Provider gives instruction on when to return	84	94	88	0.050
13	Provider performs clinical procedures according to guidelines^c^	57	60	58	0.795
15	Client participates actively in discussion and selection of method (i.e. is “empowered”)	42	47	44	0.633
Client-level FP Exit Interview (*n* = 315)					
4	Provider discusses with client which method she would prefer	35	32	34	0.635
8	Provider tailors key information to the particular needs of the specific client	93	91	92	0.546
14	Staff treats client with dignity and respect^d^	99	98	99	0.236
25	Waiting time acceptable	87	85	86	0.732

^a^For this study, the cut-off was set at 6 months

^b^Nine cases did not obtain a method and were, therefore, excluded

^c^Forty-six cases did not undergo a clinical procedure and were, therefore, excluded

^d^One case did not provide information for this indicator and was, therefore, excluded

Integration status was significantly associated with meeting three QIQ indicators. For facility-level QIQ indicators, integrated facilities were more likely to meet Indicator 18 (Facility has all [approved] methods available; no stockouts; *p* = 0.039) and Indicator 22 (Facility has received a supervisory visit in past 6 months; *p* = 0.003), compared with non-integrated facilities. For client-level QIQ indicators, integration status was significantly associated with meeting one of fifteen QIQ indicators. Clients of integrated facilities were more likely than clients of non-integrated facilities to report meeting of Indicator 1a (Look and write on client record; *p* = 0.005).

#### Tanzania

Twelve of 22 facility- and client-level QIQ indicators were met by at least half of the Tanzania SPA analytic sample (Table 5). Of seven facility-level indicators, four were met by at least half of integrated and non-integrated facilities in the sample. Of fifteen client-level QIQ indicators, eight were reported as met by clients in at least half of facilities in each integrated and non-integrated category. The sets of QIQ indicators reported by at least half of facilities and clients in Malawi and Tanzania were largely identical, though discrepancies were found (three indicators were reported by at least half of facilities or clients in one country but not another country).

**Table 5:** Percentage of non-integrated and integrated facilities meeting each QIQ Indicator (Tanzania SPA 2014–2015)

Indicator #	Description	Non-Integrated	Integrated	Total	*p*-value
Facility-level Inventory (*n* = 750)		%	%	%	
11	Provider (at facility) follows infection control procedures outlined in guidelines	61	62	62	0.921
18	Facility has all (approved) methods available; no stockouts	54	68	59	0.003
19	Facility has basic items needed for delivery of methods available through facility (sterilizing equipment, gloves, blood pressure cuff, specula, adequate lighting, water)	11	15	13	0.232
21	Facility has mechanisms to make programmatic changes based on client feedback	24	34	27	0.019
22	Facility has received a supervisory visit in past 6 months	90	95	92	0.104
23	Facility has adequate storage of contraceptives and medicines (away from water, heat, direct sunlight) on premises	42	57	47	0.002
24	Facility has state-of-the-art clinical guidelines	56	63	58	0.132
Client-level FP Observation (*n* = 1060)					
1a	Look and write on client record	72	78	75	0.236
1b	Used any visual aids	12	14	13	0.439
1c	Ensured visual and auditory privacy	75	72	74	0.662
2	Provider assures client of confidentiality	41	34	37	0.235
3	Provider asks client about reproductive intentions	34	33	33	0.867
5	Provider mentions HIV/AIDS (initiates or responds)	19	22	21	0.530
6	Provider discusses dual method use	6	8	7	0.414
9	Provider gives accurate information on the method accepted (how to use, side effects, complications)^a^	64	61	63	0.643
10	Provider gives instruction on when to return	83	82	83	0.829
13	Provider performs clinical procedures according to guidelines^b^	54	53	54	0.883
15	Client participates actively in discussion and selection of method (i.e. is “empowered”)	50	51	50	0.942
Client-level FP Exit Interview (*n* = 1059)					
4	Provider discusses with client which method she would prefer	33	37	35	0.359
8	Provider tailors key information to the particular needs of the specific client	97	95	96	0.628
14	Staff treats client with dignity and respect	97	94	95	0.106
25	Waiting time acceptable	84	71	77	0.005

^a^Thirty-seven cases did not obtain a method and were, therefore, excluded

^b^A total of 291 cases did not undergo a clinical procedure. One case reported no information for the indicator. All were excluded

Integration status was significantly associated with meeting four QIQ indicators. Compared with Malawi, integration status was positively associated with Indicator 18 (Facility has all [approved] methods available; no stockouts; *p* = 0.003) but not Indicator 22 (Facility has received a supervisory visit in past 6 months; *p* = 0.104). In addition to Indicator 18, integration status was positively associated with Indicator 21 (Facility has mechanisms to make programmatic changes based on client feedback; *p* = 0.019) and Indicator 23 (Facility has adequate storage of contraceptives and medicines [away from water, heat, direct sunlight] on premises; *p* = 0.002). At the client-level, integration status was negatively associated with Indicator 25 (Waiting time acceptable; *p* = 0.005) but not significantly associated with any other client-level QIQ indicator.

### Association between integration status and FP quality of care: multivariate analyses

#### Malawi

In Malawi (Table 6), HIV integrated facilities had two times the odds of being at or above the median in facility-level FP quality of care than non-integrated facilities in unadjusted (odds ratio [OR] = 2.18; 95% CI = 1.36, 3.50) and adjusted (OR = 2.24; 95% CI = 1.32, 3.79) facility-level models. Facilities with a private (for profit) managing authority (vs. government/public) had increased odds of being at or above the median in facility-level FP quality of care (OR = 5.42; 95% CI = 1.64, 17.91). Health post/maternity facilities had 77% reduced odds of being at or above the median in facility-level FP quality of care (OR = 0.23; 95% CI = 0.05, 0.97), compared with dispensaries. There was no significant association of urban/rural location or zone with facility-level FP quality of care. No association was found between integration status and FP quality of care assessed at the client-level (OR = 1.05; 95% CI = 0.48, 2.31).

**Table 6:** Association of integration status with FP quality of care (Malawi SPA 2013–2014)

	Facility-level				Client-level^a^	
	Unadjusted		Adjusted		Unadjusted	
	OR	(95% CI)	OR	(95% CI)	OR	(95% CI)
HIV integrated	2.18^b^	(1.36, 3.50)	2.24^b^	(1.32, 3.79)	1.05	(0.48, 2.31)
Managing Authority						
Government/Public			ref			
Private (non-profit)			5.42^b^	(1.64, 17.91)		
Private (for profit)			0.64	(0.23, 1.77)		
Facility Type						
Dispensary			ref			
Clinic			2.21	(0.67, 7.26)		
Health post/maternity			0.23^c^	(0.05, 0.97)		
Location						
Urban			ref			
Rural			1.32	(0.73, 2.40)		
Zone						
Northern			ref			
Central east			0.47	(0.16, 1.38)		
Central west			1.06	(0.43, 2.59)		
Southeast			0.63	(0.25, 1.64)		
Southwest			1.50	(0.61, 3.69)		
N	305		305		323	

^a^Indicators 9 and 13 are excluded due to smaller number of responses from non-applicable services for certain respondents

^b^*p* < 0.01

^c^*p* < 0.05

Client-level regressions account for clustering of clients in facilities

#### Tanzania

In Tanzania (Table 7), HIV integrated facilities had twice the odds of being at or above the median in facility-level FP quality of care than non-integrated facilities in unadjusted (OR = 2.26; 95% CI = 1.51, 3.37) and adjusted (OR = 2.10; 95% CI = 1.37, 3.22) facility-level models. Dispensaries had lower odds than health center/clinics to be at or above the median in facility-level FP quality of care (OR = 0.57; 95% CI = 0.38, 0.84). However, managing authority and urban/rural location were not significantly associated with the likelihood of being at or above the median in facility-level FP quality of care. An association between integration status and FP quality of care assessed at the client-level was not found (OR = 0.91; 95% CI = 0.55, 1.51).

**Table 7:** Association of integration status with FP quality of care (Tanzania SPA 2014–2015)

	Facility-level				Client-level^a^	
	Unadjusted		Adjusted		Unadjusted	
	OR	(95% CI)	OR	(95% CI)	OR	(95% CI)
HIV integrated	2.26^b^	(1.51, 3.37)	2.06^c^	(1.37, 3.22)	0.91	(0.55, 1.51)
Managing Authority						
Government/Public			ref			
Not Government/Public			0.73	(0.35, 1.53)		
Facility Type						
Health center/clinic			ref			
Dispensary			0.52^d^	(0.33, 0.83)		
Location						
Urban			ref			
Rural			0.62	(0.33, 1.14)		
N	750		750		1060	

^a^Indicators 9 and 13 are excluded due to smaller number of responses from non-applicable services for certain respondents

^b^*p* < 0.001

^c^*p* < 0.05

^d^*p* < 0.01

Client-level regressions account for clustering of clients in facilities

Region estimates now shown

## Discussion

The present study used publicly available service provision data from Malawi (2013–2014) and Tanzania (2014–2015) to evaluate whether integration of HIV services was associated with FP quality of care. Using the QIQ tool to define and measure FP quality of care, we examined whether integration status was associated with meeting several indicators (in bivariate analyses) and facility- and client-level FP quality of care (in multivariate analyses). To our knowledge, this study is the first study to match SPA measures with the majority (21 of 25) of QIQ indicators to examine service quality. We found that integration status was positively associated with facility-level FP quality of care measures in both countries, as well as a subset of facility- and client-level QIQ indicators in Malawi (*n* = 3) and Tanzania (*n* = 4).

Our facility-level bivariate and multivariate analyses found a positive association between integration status and facility-level FP quality of care. The mechanism of this relationship might be best exemplified by the consistent finding in both countries that integrated facilities were more likely than non-integrated facilities to meet criteria for Indicator 18 (Facility has all [approved] methods available; no stockouts). It is possible that HIV integrated facilities in Malawi and Tanzania benefit from strengthened or parallel supply chains implemented to scale-up antiretroviral therapy [21–23], and could efficiently receive FP commodities that might also flow through such chains. However, we have no information on facility supply chains in our sample to assess the plausibility of this explanation.

In contrast to facility-level analyses, the relationship between integration status and client-level FP quality of care in Malawi and Tanzania was less clear. Of the 15 client-level QIQ indicators, integration status was significantly associated with just one indicator in Malawi and Tanzania. Moreover, the one indicator that significantly differed by integration status in Tanzania (Waiting time acceptable) was different than Malawi (Look and write on client record) and in the opposite direction. The association between integration status and client-level FP quality of care was null for both countries. These mixed results may suggest that the most robust benefits conferred by integration of HIV services might be primarily infrastructural in nature, and that client-level outcomes that are more dependent on provider skills and capacities might be less influenced. Further research is needed to understand the consequences of integration from the provider and client perspective, and whether this finding is unique to Malawi in sub-Saharan Africa.

Our study findings must be considered along with their limitations. First, our principal data sources are two cross-sectional surveys. Therefore, we lack temporal sequence to establish a causal effect of HIV services integration on FP quality of care. Second, there are no standardized decision rules for meeting of a QIQ indicator. We set indicator criteria in accordance with the original QIQ definition, content knowledge, and data distributions of SPA measures. Consequently, our indicator criteria may be inconsistent with other studies in the literature; other criteria may be equally appropriate but result in different findings. Third, many facilities did not contribute client data through Client Observation or FP Exit Interviews. Due to the incomplete assessment of clients across all facilities, we conducted separate analyses of FP quality of care at the facility- and client-level per country, rather than a single overall analysis utilizing one dependent variable representing FP quality of care. As mentioned, the SPA data collectors would not return to facilities that had no clients visiting for the service on the day of the visit. Therefore, our sample may reflect busier facilities that have less time available for clients, and consequently perform worse on client-level indicators than facilities that are less busy and have more time to provide better quality service to FP clients. Fourth, our data-driven dichotomization of FP quality of care may limit comparison of our findings to other studies. However, the dichotomization scheme provides insight as to how integrated facilities perform relative to non-integrated facilities in the countries, according to country-specific baseline levels of FP quality of care. Finally, our analysis focused on primary and secondary health care facilities, as virtually all tertiary facilities were found to meet the criteria for integration. Results should not be interpreted for tertiary level facilities in these countries.

Our study had many strengths. We used the most recent, nationally representative data available, thereby providing valuable insight that may inform current policymaking regarding integrated FP programs. The present study is one of the few that leveraged SPA data, which is relatively underutilized given the critical need for research on topics of health system strengthening in developing countries. We used the QIQ to conduct a theory-based evaluation of FP quality of care that does not rely on single subjective measures of quality, as is common in the literature. As a result, our assessment of FP quality of care in integrated and non-integrated settings amounts to a significant contribution to the evidence base.

## Conclusion

Research on the relationship between integration of HIV services and FP quality of care is necessary to ensure that service integration results in high quality care that improves service delivery and benefits clients’ health. Using service provision data from Malawi and Tanzania, we found that integration is beneficially associated with facility-level FP quality of care. However, results were mixed at the client-level.

Our findings did not confirm concerns regarding the potential adverse consequences of HIV and FP services integration. Though research on stakeholder perspectives regarding integration implementation indicate concern that integration may overburden facilities and negatively affect quality [4], we found only one negative association indicating that integration of HIV services may negatively affect provider practice (i.e. reduced likelihood of acceptable waiting time in Tanzania). In general, our findings suggest that FP quality of care may be equivalent or superior in integrated facilities compared with non-integrated facilities in Malawi and Tanzania. Further research is needed to understand how HIV services integration may impact FP quality of care in diverse settings, and how the platform in which FP is integrated may differentially influence FP quality of care.

A French translation of this article has been included as Additional file 2.

A Portuguese translation of the abstract has been included as Additional file 3.

## Additional file

Additional file 1: **Table S1.** Description of Matched QIQ and SPA Measures in Present Study. (DOCX 15 kb)

Additional file 2: Translation of this articles into French. (PDF 361 kb)

Additional file 3: Translation of the abstract of this article into Portuguese. (PDF 115 kb)

## Abbreviations

CI: Confidence interval
FP: Family planning
OR: Odds ratio
QIQ: Quick Investigation of Quality
SPA: Service Provision Assessment
