# Supplementary material for: Quality of family planning services in HIV integrated and non-integrated health facilities in Malawi and Tanzania
Source: Reprod Health. 2019 May 29;16(Suppl 1):58. doi: 10.1186/s12978-019-0712-y (PMC6538555; doi:10.1186/s12978-019-0712-y)
Supplement: Supplementary file 1 — Table S1. Description of Matched QIQ and SPA Measures in Present Study. (DOCX 15 kb) [file 12978_2019_712_MOESM1_ESM.docx]

# **Additional File 1**

| **Table S1** Description of Matched QIQ and SPA Measures in Present Study | | | | | |
| --- | --- | --- | --- | --- | --- |
| QIQ | | SPA | | | |
| QIQ Indicator # | QIQ Indicator Description | SPA Instrument | Description of Matched SPA Measures | | Description of Dichotomous QIQ Indicators Created for Analyses |
| 1 | Provider demonstrates good counseling skills | Observation | I1A | Looked and wrote on client record | 1 = looked and wrote on client record, 0 = did not look and write on client record. |
|  |  |  | I1B | Used any visual aids | 1 = used any visual aids, 0 = did not use any visual aids. |
|  |  |  | I1C | Visual and auditory privacy ensured | 1 = ensured visual and auditory privacy, 0 = did not ensure visual and auditory privacy. |
| 2 | Provider assures client of confidentiality | Observation | Assured the client orally of confidentiality | | 1 = confidentiality orally assured, 0 = not orally assured. |
| 3 | Provider asks client about reproductive intentions | Observation | Desire for a child or more children | | 1 = desire for a child OR birth timing asked, 0 = neither asked. |
|  |  |  | Desired timing for birth of next child | |  |
| 4 | Provider discusses with client which method she would prefer | Client Interview | [A composite of measures asking about whether client thought about FP method prior to the visit.] | | 1 = provider talked to client about method of interest  0 = provider did not talk to client about method of interest  0 = OR client did not think about switching method  0 = OR client did not think about what method she wanted to use  0 = OR client stopped using method (elective – no problems). |
| 5 | Provider mentions HIV / AIDS (initiates or responds) | Observation | Client's perceived risk of STIs / HIV | | 1 = client’s perceived risk of STIs/HIV OR use of condoms discussed, 0 = neither discussed. |
|  |  |  | Use of condoms to prevent STIs/HIV | |  |
| 6 | Provider discusses dual method use | Observation | Using condoms along with another method (dual method) to prevent both | | 1 = dual method use discussed, 0 = not discussed. |
| 7 | Provider treats client with respect/dignity | No match available. | | | |
| 8 | Provider tailors key information to the particular needs of the specific client | Client Interview | Amount of explanation you received about the problem or treatment | | 1 = no problem with explanation received, 0 = minor problem, major problem, or don’t know. |
| 9 | Provider gives accurate information on the method accepted (how to use, side effects, complications) | Observation | [A composite of itemized tasks of information to be communicated per method.] | | 1 = at or above the median in required information communicated for at least one selected procedure provided or prescribed, 0 = below median of required information communicated for all selected procedures. |
| 10 | Provider gives instruction on when to return | Observation | Discussed a return visit | | 1 = discussed a return visit, 0 = did not. |
| 11 | Provider follows infection control procedures outlined in guidelines | Inventory | [A composite of standard precautions and conditions for client examination (e.g. running water, hand-washing soap).] | | 1 = at or above the median in number of observed infection prevention precaution measures, 0 = below the median. |
| 12 | Provider recognizes / identifies contraindications consistent with guidelines | No match available. | | | |
| 13 | Provider performs clinical procedures according to guidelines | Observation | [A composite of itemized clinical tasks to be completed per clinical procedure.] | | 1 = at or above median in required tasks before / during / after procedure for at least one procedure, 0 = below median for all selected procedures. |
| 14 | Staff treats client with dignity and respect | Client Interview | How the staff treated you | | 1 = no problem with how staff treated you, 0 = minor problem, major problem, or don’t know. |
| 15 | Client participates actively in discussion and selection of method (i.e. is “empowered”) | Observation | Client expressed concerns about method, or asked questions about method, including possible side effects of method. | | 1 = client expressed concerns […], including possible side effects of method, 0 = did not. |
| 16 | Client receives her method of choice | No match available. | | | |
| 17 | Client believes the provider will keep her information confidential | No match available. | | | |
| 18 | Facility has all (approved) methods available; no stockouts | Inventory | [A composite of methods provided and in-stock (i.e. at least one valid contraceptive commodity observed per method).] | | 1 = at least 4 methods provided and in-stock (at least one valid), 0 = else. |
| 19 | Facility has basic items needed for delivery of methods available through facility (sterilizing equipment, gloves, blood pressure cuff, specula, adequate lighting, water) | Inventory | [A composite of basic equipment and supplies observed in facilities.] | | For non-IUD: 1 = blood pressure device (digital or manual) and examination light observed, 0 = else.  For IUD: 1 = blood pressure device (digital or manual), examination light, and speculum (any size) observed, 0 = else. |
| 20 | Facility offers privacy for pelvic exam / IUD insertion (no one can see) | Excluded. | | | |
| 21 | Facility has mechanisms to make programmatic changes based on client feedback | Inventory | [A composite of measures regarding implementation of a system to determine client feedback and whether there is a procedure to review feedback.] | | 1 = yes, procedure for reviewing or reporting on clients’ opinion  0 = no procedure to review or report on client’ opinions  0 = OR no system to determine clients’ opinion  0= OR don't know whether there is procedure for reviewing or reporting on clients’ opinion  0 = OR yes, have a system to determine clients’ opinion but missing on whether there is procedure for reviewing or reporting on clients’ opinion |
| 22 | Facility has received a supervisory visit in past __ months | Inventory | When was the last time a supervisor from outside this facility came here on a supervisory visit? | | 1 = yes, visit within 6 months, 0 = yes, visit over 6 months ago  0 = OR no external supervision. |

*QIQ* Quick Investigation of Quality

*SPA* Service Provision Assessment
