# Supplementary material for: Quality of family planning services in HIV integrated and non-integrated health facilities in Malawi and Tanzania
Source: Reprod Health. 2019 May 29;16(Suppl 1):58. doi: 10.1186/s12978-019-0712-y (PMC6538555; doi:10.1186/s12978-019-0712-y)
Supplement: Supplementary file 2 — Translation of this articles into French. (PDF 361 kb) [file 12978_2019_712_MOESM2_ESM.pdf]

Qualité des services de planning familial dans les établissements de santé proposant des services intégrés et non intégrés de traitement du VIH au Malawi et en Tanzanie

Michael A. Close[1] , Janine Barden-O'Fallon[1*] , Carolina Mejia[1]

[1]Carolina Population Center, Health Behavior Department, Gillings School of Global Public Health,
University of North Carolina at Chapel Hill, Chapel Hill, NC, USA

MC: mclose@email.unc.edu
JB: bardenof@email.unc.edu
CM cmejia@unc.edu

* Auteur correspondant : Janine Barden-O'Fallon

Résumé

Contexte

L'intégration des services liés au planning familial (PF) et au VIH est courante en Afrique subsaharienne. Peu de recherches ont examiné en quoi la qualité des soins de PF diffère entre les établissements avec services intégrés et non intégrés. À l'aide de données nationales représentatives du Malawi et de la Tanzanie, nous avons examiné la manière dont l'intégration du VIH était associée à la qualité des soins de PF.

Méthodes

Les données proviennent des évaluations des prestations de services (SPA) du Malawi (2013–2014) et de la Tanzanie (2014–2015). L'échantillon analytique a été limité aux structures de niveau inférieur au Malawi (n = 305) et en Tanzanie (n = 750) qui offraient des services de PF. Dans l'investigation rapide de la qualité (QIQ), nous avons fait correspondre les mesures de SPA avec les indicateurs de la qualité des soins de PF. Nous avons effectué des analyses bidimensionnelles et multivariées de 22 indicateurs QIQ afin d'examiner le lien entre l'état d'intégration et les indicateurs QIQ individuels et la qualité globale des soins de PF aux niveaux de l'établissement et des patientes.

Résultats

La prévalence de l'intégration des services liés VIH au Malawi (39 %) et en Tanzanie (38 %) était similaire. L'intégration des services liés au VIH était associée de manière significative ($p < 0{,}05$) aux indicateurs QIQ au Malawi (n = 3) et en Tanzanie (n = 4). À l'exception d'une association négative en Tanzanie, toutes les autres associations étaient positives. Au niveau de l'établissement, l'intégration des services liés au VIH présentait la probabilité accrue d'être au moins égale à la moyenne de la qualité des soins de PF au Malawi (rapports de cote ajusté (RC) = 2,24 ; intervalle de confiance (IC) à 95 % = 1,32, 3,79) et Tanzanie (RC ajusté = 2,10 ; IC 95 % = 1,37, 3,22). Au niveau de la patiente, l'intégration des services liés au VIH n'était pas associée à la qualité des soins de PF dans les deux pays.

Conclusion

D'après des échantillons prélevés au Malawi et en Tanzanie, l'intégration des services liés au VIH semble être associée de manière bénéfique à la qualité des soins de PF. En utilisant un éventail d'indicateurs de la qualité des soins de PF, nous avons trouvé peu de preuves pour étayer les préoccupations selon lesquelles l'intégration des services liés au VIH pourrait mettre à rude épreuve les établissements et les prestataires et avoir un impact négatif sur les résultats en termes de qualité. Cela semble plutôt renforcer la prestation de services de PF en augmentant la probabilité que les produits de PF soient stockés et que d'autres indicateurs de qualité au niveau des établissements soient atteints, éventuellement par le biais des chaînes d'approvisionnement liées aux services de prise en charge du VIH. Des recherches supplémentaires sont nécessaires pour évaluer les résultats de la qualité des soins du PF parmi les diverses plateformes d'intégration du PF trouvées en Afrique subsaharienne.

Mots clés : Qualité du service, Indice de qualité, Intégration, Planning familial, VIH, SPA, QIQ, Malawi, Tanzanie

Contexte

L'intégration des services de planning familial (PF) et liés au VIH est une tendance à long terme des systèmes de santé en Afrique subsaharienne [1]. Bien que le PF ait été intégré à une multitude d'autres services de santé (services de santé maternelle, néonatale et infantile, par exemple) [2], les services liés au VIH constituent une plateforme d'intégration de premier plan en raison de la prévalence du VIH et du SIDA dans la région [1]. Nous définissons l'intégration de service comme la prestation de deux types de services de santé différents dans le même établissement, bien que diverses définitions plus précises de l'intégration soient présentes dans la littérature existante [1,3]. La prestation de services intégrés est devenue de plus en plus répandue à mesure que la recherche s'approfondissait sur ses effets potentiellement favorables et bénéficiait du soutien des parties prenantes locales [4].

La littérature sur les effets de l'intégration des services de PF et de VIH sur les résultats au niveau de l'établissement, du prestataire et de la patiente est largement positive, bien que peu concluante. L'intégration des services de PF et de VIH a été associée à des résultats cliniques bénéfiques (par exemple, la prévention des naissances séropositives non souhaitées) [5,6], des résultats en matière de prestation de services (par exemple, une meilleure utilisation des services) [7] et rentabilité (par exemple, réduction des coûts liés à la prévention des infections de VIH chez le nourrisson) [1,8]. Cependant, les problèmes d’infrastructure et de logistique liés à l’intégration des services de santé [4] sont susceptibles d’affecter négativement la qualité du service de base [9] et de diminuer l’expertise des prestataires.[10].

Malgré la prédominance des services intégrés de PF et de VIH, les recherches sur l'impact de la programmation intégrée sur la qualité des soins de PF sont rares. Le maintien de la qualité des soins de PF est essentiel pour des résultats positifs en matière de santé des patientes et pour l’adhésion à une approche de PF basée sur les droits en matière de reproduction. Le cadre de qualité des soins

Bruce/Jain, qui a guidé la conception et la prestation de services de planning familial pendant plus de deux décennies, définit six éléments essentiels qui constituent la qualité des soins de PF : le choix des méthodes, les informations fournies aux utilisateurs, les compétences techniques, les relations interpersonnelles, les mécanismes de suivi ou de continuité, et la gamme de services appropriée [11]. La nature multidimensionnelle de la qualité des soins de PF proposée par le cadre de Bruce/Jain nécessite une mesure au niveau de l'établissement (par exemple, disponibilité des méthodes de PF), au niveau du prestataire (par exemple, le respect des directives de contrôle des infections) et au niveau de la patiente (par exemple méthode de PF préférée) pour saisir au mieux de la qualité des soins de PF.

Les quelques études disponibles sur la qualité des soins de PF dans les établissements ayant intégré les services de soins liés au VIH sont limitées par des préoccupations méthodologiques. Dans une analyse des services de PF et de VIH intégrés menée à travers le monde, Spaulding et ses collègues[12] ont identifié quatre études [13–16] sur la « qualité des services », bien que la plupart de ces recherches soient tirées de la littérature grise (documentation parallèle) dont les plans d'étude sont insuffisamment détaillés ou insuffisants [14–16] ou repose sur des informations déclarées par le prestataire de soins, telles que les connaissances et les comportements [16], utilisés comme substituts pour assurer la qualité des soins de PF. Pour acquérir une compréhension complète de la qualité des soins de PF, une approche d'analyse basée sur une théorie comprenant des indicateurs de qualité au niveau de l'établissement, du prestataire et de la patiente est nécessaire pour une prise de décision éclairée basée sur des éléments probants.

La présente étude visait à renforcer les connaissances en utilisant plusieurs indicateurs objectifs issus d'un outil de mesure de la qualité des soins de PF, fondé sur la théorie, Quick Investigation of Quality (QIQ), qui permet d'évaluer la qualité des soins de PF dans les établissements ayant intégré ou non des services liés au VIH, en utilisant les données tirées de l'évaluation SPA au Malawi (2013–2014) et en Tanzanie (2014–2015). Les objectifs spécifiques de cette étude étaient d'évaluer le niveau de qualité des

soins de PF dans les établissements ayant intégré ou pas des services liés au VIH, de comparer la qualité des soins de PF entre ces établissements, et enfin de déterminer le degré d'intégration associé à la qualité des soins de PF lors du contrôle des autres caractéristiques de chaque établissement. Nous avons émis l'hypothèse selon laquelle il existe des différences dans la qualité de la prestation de services de PF entre les établissements ayant intégré des services liés au VIH et les établissements n'ayant pas intégré ces services.

Méthodes

Plan de l'étude et sources de données

Nous avons mené une étude rétrospective transversale basée sur des ensembles de données secondaires de SPA récentes menées au Malawi (2013–2014) et en Tanzanie (2014–2015). Le but du processus SPA est d'évaluer la disponibilité et la qualité des services de santé de base et essentiels pour identifier les lacunes et comparer les résultats des systèmes de santé [17,18]. Quatre types d'instruments de collecte de données sont utilisés pour comprendre les caractéristiques pertinentes au niveau de l'établissement, du prestataire de soins et de la patiente : Questionnaire d'inventaire des établissements, questionnaire d'entretien avec le prestataire de soins de santé, protocoles d'observation pour certains services de santé (y compris PF) et questionnaires d'entretien de sortie pour certaines patientes et certains soignants (y compris les patientes du PF). En résumé, ces outils de collecte de données fournissent un aperçu complet de la situation d'un large éventail de services de santé de base et essentiels, y compris ceux liés au PF et au VIH.

Nous avons utilisé les données collectées à partir du questionnaire d'inventaire des établissements, du protocole d'observation du PF et du questionnaire d'entretien à la sortie de la patiente du PF. Les méthodologies de collecte de données pour le Malawi et la Tanzanie étaient en grande partie identiques [17,18]. Une équipe d'agent de collecte des données s'est rendue dans chaque établissement pour

remettre des questionnaires et appliquer des protocoles d'observation. Pour le questionnaire d'inventaire des établissements, un agent de collecte des données s'est rapproché des membres compétents du personnel ayant des informations pertinentes pour répondre aux questions de chaque section. Pour le protocole d'observation du PF, les agents de collecte des données ont été priés d'observer un maximum de cinq patientes pour chaque prestataire de soins, avec un maximum de 15 observations par service et par établissement. Si plusieurs patientes éligibles au PF étaient présentes et attendaient un rendez-vous, les enquêteurs ont cherché à sélectionner deux nouvelles patientes pour chaque patiente suivie. Chaque patiente ayant fait l'objet d'une consultation a ensuite été approchée pour répondre au questionnaire de l'entretien de départ d'une patiente du PF. Si le service n'était pas offert le jour de l'arrivée des enquêteurs, une nouvelle visite était organisée pour appliquer le protocole d'observation et effectuer les entretiens correspondants. Cependant, aucune visite de retour n'a été effectuée si le service était offert ce jour-là, mais aucune patiente n'est venue pour bénéficier du service. Par conséquent, tous les établissements de l'échantillon ne disposent pas des données du protocole d'observation de PF et de l'entretien de départ de la patiente du PF. Des détails supplémentaires sur le processus de SPA sont rapportés ailleurs [17–19].

Échantillon choisi

Les établissements inclus dans l'échantillon offraient tous les services de PF, comme indiqué dans la section Disponibilité générale des services de l'inventaire des établissements. Dans la présente étude, nous définissons l'intégration du VIH par un établissement offrant des services de PF outre soit « des services de prescription d'antirétroviraux pour le VIH/SIDA ou de suivi d'un traitement antirétroviral », soit des « services de soins et de soutien pour le VIH/SIDA, notamment le traitement des infections opportunistes et la fourniture de soins palliatifs ». Les établissements étaient considérés comme « non intégrés » s'ils offraient des services de conseil et de dépistage du VIH, mais aucune des deux catégories de services de soins et de soutien liés au VIH. (Notez que le conseil et le dépistage du VIH sont une

pratique courante dans les établissements offrant des services de PF, 85 % au Malawi et 98 % en Tanzanie offraient également des services de conseil et de dépistage du VIH et presque tous les établissements inclus dans l'analyse au Malawi (119/121) et La Tanzanie (394/396) fournissaient au moins une méthode de contraception réversible à action prolongée (LARC). Il est important de noter que les établissements intégrés et non intégrés offraient une variété de services de soins de santé primaires, tels que des soins prénataux et des services de santé dédiés à l'enfant, en plus du PF. La Figure 1 présente un organigramme d'étude de la stratégie exemple.

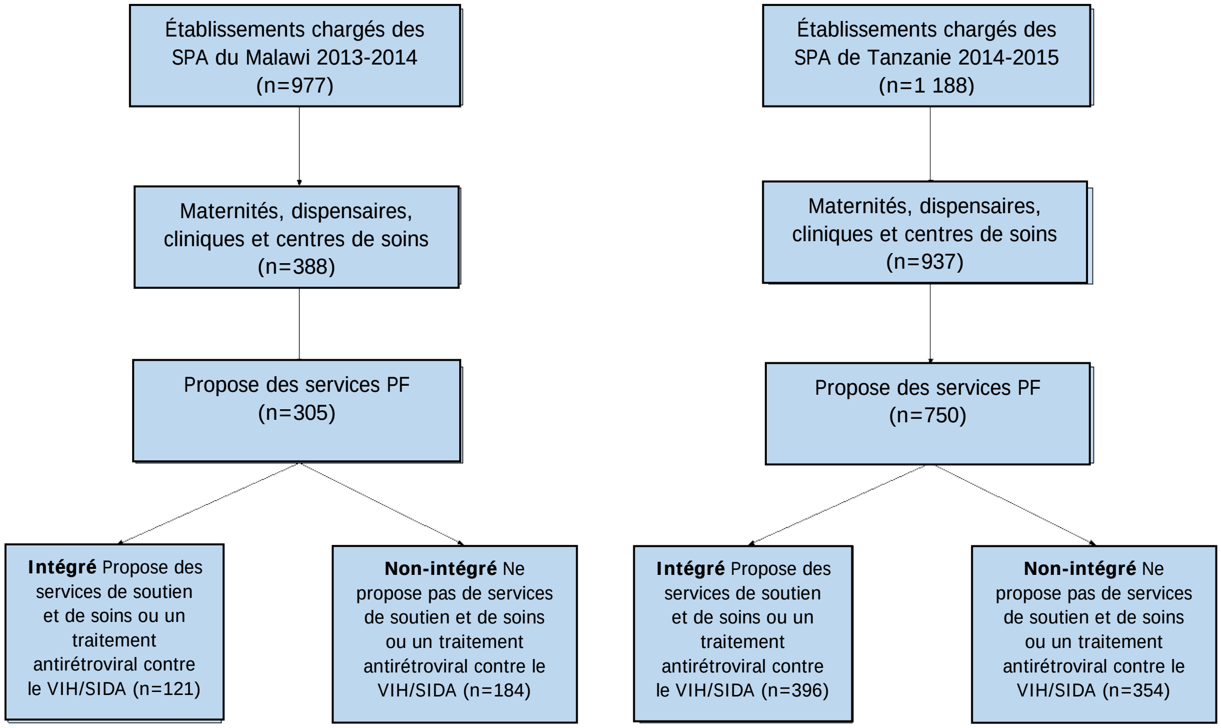

Figure 1. Étude sur l'inclusion des établissements tirée des données des processus de SPA au Malawi 2013-2014 et en Tanzanie 2014-2015

Malawi

L'évaluation SPA du Malawi a permis de recenser tous les établissements du secteur officiel du pays. Au Malawi, 977 établissements sur 1060 (92 %) ont été évalués et inclus dans l'ensemble de données de SPA. Les établissements du panel de sondage non évalués étaient les suivants : refus (3 %), fermés/pas

encore opérationnels (2 %), aucun répondant disponible (1 %) et inaccessibilité (2 %). La stratification par type d'établissement a montré que, sur 505 hôpitaux et centres de santé offrant des services de PF, seulement 20 (4 %) n'étaient pas intégrés. Étant donné que l'intégration dans ces types d'établissements est quasiment totale (100 %), ils ont été exclus de l'analyse, ce qui a laissé 388 maternités, dispensaires, cliniques et postes de santé. Sur les 388 établissements, 305 (79 %) offrent des services de PF. Parmi les établissements offrant des services de PF, 121 (40 %) offraient également « des services de suivi des ordonnances antirétrovirales pour le VIH/SIDA ou de suivi des traitements antirétroviraux » ou « des services de prise en charge du VIH/SIDA, y compris le traitement des infections opportunistes et la fourniture de soins palliatifs ». Par conséquent, l'échantillon analytique au niveau de l'établissement (n = 305) avait une plus faible proportion d'établissements qui étaient intégrés au panel d'établissement offrant des services liés VIH (40 %) par rapport aux établissements non intégrés (60 %). Sur les 305 établissements, 108 ont eu des observations de patientes de PF (n = 323) et des entretiens de sortie des patientes du PF (n = 315) et constituent l'échantillon analytique au niveau de la patiente.

Tanzanie

Le processus de SPA de la Tanzanie était une enquête par sondage basée sur les probabilités et représentative au niveau national de tous les établissements du secteur officiel du pays. En Tanzanie, 1188 établissements sur 1200 (99 %) faisant partie du panel de sondage ont été évalués et inclus dans l'ensemble de données du processus de SPA. Les établissements faisant partie du panel de sondage mais non évalués (1 %) avaient refusé de répondre (n = 7), étaient fermés/pas encore opérationnels (n = 4) et inaccessibles (n = 1). La stratification par type d'établissement a montré que sur les 183 hôpitaux offrant des services de PF, seuls huit (4 %) n'étaient pas intégrés au panel d'établissements offrant des services de prise en charge du VIH. Comme au Malawi, l'intégration atteignait presque 100 % à ce niveau de prestation de services. 937 centres de santé, cliniques et dispensaires ont été exclus de l'analyse. Parmi ces établissements, 750 (80 %) offraient des services de PF. Parmi les établissements offrant des services

de PF, 396 (53 %) offraient également « des services de suivi des ordonnances antirétrovirales pour le VIH/SIDA ou de suivi des traitements antirétroviraux » ou « des services de prise en charge du VIH/SIDA, y compris le traitement des infections opportunistes et la fourniture de soins palliatifs ». Par conséquent, l'échantillon analytique au niveau de chaque établissement (n = 750) était à peu près également divisé en deux catégories : intégré (53 %) et non intégré (47 %). Sur les 750 établissements, 365 ont eu des observations de patientes de PF (n = 1060) et des entretiens de sortie des patientes de PF (n = 1059) et constituent l'échantillon analytique au niveau de la patiente.

Mesures

Indicateurs

Les indicateurs QIQ ont été mis en correspondance avec les mesures SPA pour créer des indicateurs de la qualité des soins de PF (Tableau 1) [20]. Le processus de QIQ a été développé par le projet d'évaluation MEASURE afin de fournir une méthodologie rapide et peu coûteuse pouvant être utilisée pour mesurer systématiquement la qualité des soins dans les programmes de planning familial en clinique et les services de santé génésique connexes. Le processus de QIQ comprend 25 indicateurs qui mesurent cinq des six éléments du cadre de qualité des soins de Bruce/Jain : choix des méthodes, informations, compétences techniques, relations interpersonnelles et suivi [11,20]. La méthodologie de collecte de données était similaire à celle du processus de SPA en ce sens qu'un questionnaire de vérification des établissements, un protocole d'observation du PF et un entretien à la sortie de la patiente ont été mis en place pour évaluer plusieurs niveaux de qualité des soins de PF.

Nous avons associé les mesures SPA à 21 des 25 indicateurs QIQ d'origine. Cependant, nous avons exclu l'un de ces indicateurs QIQ (indicateur 20) en raison du très petit nombre de patientes des échantillons analytiques du Malawi et de la Tanzanie contenant des informations sur cet indicateur. Puisque nous avons traité l'indicateur 1 comme trois sous-indicateurs distincts dans les analyses, 22 indicateurs de QIQ ont été utilisés dans nos analyses. Chaque indicateur a été considéré comme une variable

dichotomique. Un fichier supplémentaire présente la cartographie SPA et la dichotomisation de chaque indicateur QIQ [voir le Fichier supplémentaire 1, Tableau S1].

Analyse des données

Des analyses bivariées ont été effectuées pour comparer chaque indicateur QIQ selon le statut VIH intégré ou non intégré. Le test du chi carré de Pearson a été utilisé pour examiner si la répartition de chaque indicateur QIQ différait de manière significative en fonction du statut d'intégration. Les résultats sont présentés sous forme de pourcentages.

Des analyses de régression logistique ont été utilisées pour déterminer si le statut d'intégration était associé à la qualité des soins de PF. Nous avons estimé des modèles non ajustés de la relation entre l'état d'intégration et la qualité des soins de PF, à la fois au niveau de l'établissement et de la patiente. En outre, nous avons estimé des modèles ajustés au niveau du contrôle de l'établissement pour l'autorité de gestion (Malawi : 1 = gouvernement/secteur public, 2 = secteur privé [à but non lucratif] et 3 = privé [à but lucratif] ; Tanzanie : 1 = gouvernement/secteur public , 0 = pas du gouvernement/secteur public), type d'établissement (Malawi : 1 = dispensaire, 2 = clinique, 3 = poste de santé/maternité ; Tanzanie : 1 = centre de santé/clinique, 2 = dispensaire), zone (pour le Malawi) ou région (pour la Tanzanie) et la localisation urbaine/rurale (1 = rural, 0 = urbain). Les variables dépendantes de la qualité des soins de PF pour les modèles au niveau de l'établissement et de la patiente étaient la somme des scores des indicateurs QIQ respectifs de l'établissement et de la patiente dichotomisés à la médiane. La somme des scores comprend tous les indicateurs relatifs aux établissements et aux patientes, à l'exception de l'Indicateur 9 (le prestataire donne des informations précises sur la méthode acceptée [mode d'utilisation, effets secondaires, complications]) et de l'Indicateur 13 (le prestataire effectue les procédures cliniques conformément aux lignes directrices), car peu de répondants ont reçu une offre de service pertinente et n'avaient donc aucune information sur l'indicateur. La variable dépendante de la qualité des soins de PF au niveau de l'établissement

comprenait sept indicateurs et le score total variait de 0 à 7 pour le Malawi (médiane = 4, écart type [SD] = 1,64) et pour la Tanzanie (médiane = 4, SD = 1,39). La variable dépendante de la qualité des soins de PF au niveau de la patiente (avec des indicateurs tirés d'observations et d'entretiens à la sortie de la patiente) comportait 13 indicateurs et le score médian variait de 1 à 13 pour le Malawi (médiane = 7, SD = 2,03) et la Tanzanie (médiane = 7, SD = 2,04).

Les analyses ont été pondérées pour le panel d'échantillon (Tanzanie) et le panel de non-réponses (Malawi) afin de compenser toute sur-représentation ou sous-représentation du type d'établissement dans les données. Dans les modèles au niveau de la patiente, nous avons spécifié l'établissement en tant qu'unité d'échantillon principale permettant d'ajuster les écarts types pour le regroupement des patientes au sein des établissements. Un alpha bilatéral de 0,05 a été défini à des fins de pertinence statistique. Toutes les analyses ont été effectuées sous Stata 15.0 (College Station, TX, États-Unis).

Résultats

Répartition des établissements par pays

Malawi

Sur les 305 établissements inclus dans l'échantillon analytique pondéré de SPA au Malawi, 39 % répondaient aux critères d'intégration des services liés au VIH en offrant des services de PF et au moins un des deux services de prise en charge et de soutien liés au VIH. Le Tableau 2 présente les caractéristiques des établissements de l'échantillon du Malawi par statut d'intégration. L'autorité de gestion des établissements intégrés et non intégrés ne différait pas sensiblement : une majorité de l'échantillon signalait une autorité de gestion privée (à but lucratif) (61 %). La plupart des établissements intégrés et non intégrés étaient des cliniques (80 %), bien qu'une proportion plus élevée d'établissements intégrés étaient des dispensaires (18 %) par rapport aux établissements non intégrés (10 %). Bien que l'emplacement d'un établissement en milieu urbain ou rural ne diffère pas de manière

significative en fonction du statut d'intégration (p = 0,156), une proportion significativement plus élevée (p = 0,006) d'établissements intégrés se trouve concentrée dans le nord (17 %) et le sud-est (24 %), par rapport aux établissements non intégrés.

Tanzanie

Parmi les 750 établissements inclus dans l'échantillon analytique SPA pour la Tanzanie, 38 % étaient intégrés aux services de prise en charge du VIH, tels qu'ils sont définis en offrant des services de PF et au moins l'un des deux services de soins et de soutien liés au VIH. Le Tableau 3 présente les caractéristiques des établissements en Tanzanie. Quasiment tous les établissements de l'échantillon ont indiqué être gérés par une autorité de gestion publique/gouvernementale (88 %) et être présents dans une région rurale (83 %). La plupart des établissements intégrés (76 %) et non intégrés (95 %) de l'échantillon étaient des dispensaires. Cependant, une proportion significativement plus élevée d'établissements intégrés (p <0,0001) étaient des centres de santé ou des cliniques (24 %) par rapport aux établissements non intégrés (5 %).

Répartition des établissements répondant aux indicateurs QIQ, par statut d'intégration : analyses à deux variables

Malawi

Au moins la moitié des établissements et des patientes de 11 des 22 indicateurs QIQ ont été satisfaites dans chaque catégorie d'intégration de l'échantillon analytique du Malawi (Tableau 4). Sur sept indicateurs au niveau des établissements, seuls trois ont été atteints par au moins la moitié des établissements de chaque catégorie Intégré et Non intégré. Sur quinze indicateurs QIQ au niveau des patientes, au moins la moitié des patientes de huit catégories d'établissements intégrés et non intégrés ont répondu à huit.

Le statut d'intégration était associé de manière significative au respect de trois indicateurs QIQ. Pour les indicateurs QIQ au niveau de l'établissement, les établissements intégrés étaient plus susceptibles de respecter l'Indicateur 18 (Toutes les méthodes [approuvées] sont disponibles ; aucune rupture de stock ; p = 0,039) et l'Indicateur 22 (L'établissement a été inspecté au cours des 6 derniers mois ; p = 0,003), par rapport aux établissements sans services intégrés. Pour les indicateurs QIQ au niveau de la patiente, le statut d'intégration était fortement associé à la satisfaction de l'un des quinze indicateurs QIQ. Les patientes des établissements intégrés étaient plus susceptibles que les patientes des établissements non intégrés de répondre aux critères de l'Indicateur 1a (Consultation et annotation du dossier de patiente ; p = 0,005).

Tanzanie

Douze des 22 indicateurs QIQ au niveau de l'établissement et de la patiente ont été satisfaits par au moins la moitié de l'échantillon analytique de SPA en Tanzanie (Tableau 5). Quatre des sept indicateurs relatifs aux établissements ont été satisfaits par au moins la moitié des établissements intégrés et non intégrés de l'échantillon. Sur quinze indicateurs QIQ au niveau des patientes, huit ont été déclarés satisfaits par les patientes dans au moins la moitié des établissements, dans chaque catégorie Intégré et Non intégré. Les ensembles d'indicateurs QIQ signalés par au moins la moitié des établissements et des patientes au Malawi et en Tanzanie étaient en grande partie identiques, bien que des divergences aient été constatées (trois indicateurs ont été signalés par au moins la moitié des établissements ou des patientes dans un pays mais pas dans l'autre).

Le statut d'intégration était associé de manière significative au respect de quatre indicateurs QIQ. Comparativement au Malawi, le statut d'intégration était positivement associé à l'Indicateur 18 (L'établissement dispose de toutes les méthodes [approuvées] ; pas de rupture de stock ; p = 0,003) mais pas à l'Indicateur 22 (L'établissement a fait l'objet d'une visite de contrôle au cours des 6 derniers mois ;

p = 0,104). Outre l'Indicateur 18, le statut d'intégration était positivement associé à l'Indicateur 21 (L'établissement dispose de mécanismes pour effectuer des modifications programmatiques en fonction des retours des patientes ; *p* = 0,019) et de l'Indicateur 23 (L'établissement dispose d'un lieu de stockage adéquat des contraceptifs et des médicaments [à l'abri de l'eau, de la chaleur, de la lumière directe du soleil] dans ses locaux ; *p* = 0,002). Au niveau de la patiente, le statut d'intégration était associé négativement à l'Indicateur 25 (Temps d'attente acceptable ; *p* = 0,005) mais n'était pas associé de manière significative à aucun autre indicateur QIQ au niveau de la patiente.

Association entre le statut d'intégration et la qualité des soins de PF : analyses à plusieurs variables

Malawi

Au Malawi (Tableau 6), la qualité des soins fournis par les établissements de soins de santé au niveau de la maternité était deux fois plus grande que celle des établissements non intégrés sur des modèles non ajustés (rapports de cote [RC] = 2,18 ; IC 95 % = 1,36, 3,50) et ajustés (RC = 2,24 ; IC à 95 % = 1,32, 3,79) au niveau de l'établissement. Les établissements dotés d'une autorité de gestion privée (à but lucratif) (par rapport au gouvernement/secteur public) avaient plus de chances d'afficher des réponses égales ou supérieures à la médiane de qualité des soins de PF au niveau de l'établissement (RC = 5,42 ; IC 95 % = 1,64, 17,91). Les postes de santé/maternités avaient 77 % de chances en moins de présenter des valeurs égales ou supérieures à la médiane de qualité des soins de PF (RC = 0,23 ; IC 95 % = 0,05, 0,97), comparativement aux dispensaires. Il n'y avait pas d'association significative entre l'emplacement ou la zone urbaine/rurale et la qualité des soins en PF au niveau de l'établissement. Aucune association n'a été constatée entre le statut d'intégration et la qualité des soins de PF évalués au niveau des patientes (RC = 1,05 ; IC 95 % = 0,48, 2,31).

Tanzanie

En Tanzanie (Tableau 7), les établissements intégrés aux services de prise en charge du VIH avaient deux fois plus de chances de présenter des valeurs au moins égales à la médiane de la qualité des soins de PF par établissement, par rapport aux établissements non intégrés sur des modèles non ajustés (RC = 2,26 ; IC 95 % = 1,51, 3,37) et ajustés (RC = 2,10 ; IC à 95 % = 1,37, 3,22) au niveau de l'établissement. Les dispensaires avaient moins de chances que les centres de santé/cliniques d'afficher des valeurs égales ou supérieures à la médiane de la qualité des soins de PF au niveau de l'établissement (RC = 0,57 ; IC 95 % = 0,38, 0,84). Cependant, l'autorité de gestion et l'emplacement en zone urbaine/rurale n'étaient pas significativement associés à la probabilité d'atteindre ou de dépasser la médiane de la qualité des soins en PF au niveau de l'établissement. Aucun lien entre le statut d'intégration et la qualité des soins de PF évalués au niveau des patientes n'a été trouvé (RC = 0,91 ; IC 95 % = 0,55, 1,51).

Discussion

La présente étude a utilisé des données de prestation de services accessibles au public au Malawi (2013-2014) et en Tanzanie (2014-2015) pour évaluer si l'intégration des services liés au VIH était associée à la qualité des soins de PF. À l'aide de l'outil QIQ pour définir et mesurer la qualité des soins de PF, nous avons examiné si le statut d'intégration était associé à la satisfaction de plusieurs indicateurs (analyses bivariées) et de la qualité des soins de PF aux niveaux des établissements et des patientes (analyses multivariées). À notre connaissance, cette étude est la première étude à faire correspondre les mesures de SPA à la majorité (21 sur 25) des indicateurs QIQ pour évaluer la qualité de service. Nous avons constaté que le statut d'intégration était positivement associé aux mesures de qualité des soins de PF au niveau de l'établissement dans les deux pays, ainsi qu'à un sous-ensemble d'indicateurs QIQ au niveau de l'établissement et des patientes au Malawi (n = 3) et en Tanzanie (n = 4).

Nos analyses bivariées et multivariées au niveau de l'établissement ont mis en évidence une association positive entre le statut d'intégration et la qualité des soins en PF au niveau de l'établissement. Le mécanisme de cette relation pourrait être mieux illustré par la conclusion constante dans les deux pays selon laquelle les établissements intégrés étaient plus susceptibles que les établissements non intégrés de respecter les critères de l'Indicateur 18 (L'établissement dispose de toutes les méthodes [approuvées] disponibles ; aucune rupture de stock). Il est possible que les établissements intégrés aux services de prise en charge du VIH au Malawi et en Tanzanie bénéficient de chaînes d'approvisionnement renforcées ou parallèles mises en place pour développer le traitement antirétroviral [21–23] et pourraient efficacement recevoir des produits de PF qui pourraient également transiter par ces chaînes. Cependant, nous ne disposons d'aucune information sur les chaînes d'approvisionnement des établissements de notre échantillon pour évaluer la plausibilité de cette explication.

Contrairement aux analyses au niveau des établissements, la relation entre le statut d'intégration et la qualité des soins de PF au niveau des patientes au Malawi et en Tanzanie était moins claire. Sur les 15 indicateurs QIQ au niveau des patientes, le statut d'intégration était principalement associé à un seul indicateur au Malawi et en Tanzanie. En outre, le seul indicateur qui différait de manière significative en fonction du statut d'intégration en Tanzanie (délai d'attente acceptable) était différent de celui du Malawi (Consultation et annotation du dossier de patiente) et inversement. L'association entre le statut d'intégration et la qualité des soins de PF au niveau des patientes était nulle pour les deux pays. Ces résultats mitigés peuvent suggérer que les avantages les plus solides conférés par l'intégration des services de VIH pourraient être principalement de nature infrastructurelle et que les résultats au niveau des patientes qui dépendent davantage des compétences et des capacités du prestataire pourraient être moins influencés. Des recherches supplémentaires sont nécessaires pour comprendre les

conséquences de l'intégration du point de vue du prestataire de soins et des patientes et déterminer si cette constatation est propre au Malawi en Afrique subsaharienne.

Les résultats de notre étude doivent être considérés en tenant compte de leurs limites. Premièrement, nos principales sources de données sont deux enquêtes transversales. Par conséquent, nous manquons de séquence temporelle pour établir un effet causal de l'intégration des services de VIH sur la qualité des soins de PF. Deuxièmement, il n'y a pas de règles de décision standardisées pour la satisfaction d'un indicateur QIQ. Nous avons défini les critères d'indicateurs sur la base de la définition QIQ d'origine, de la connaissance du contenu et des répartitions de données des mesures de SPA. Par conséquent, nos critères d'indicateurs peuvent être incompatibles avec d'autres études dans la littérature existante ; d'autres critères peuvent être tout aussi appropriés, mais aboutir à des résultats différents. Troisièmement, de nombreux établissements n’ont pas fourni de données sur les patientes par le biais d’observation des patientes ou d’entretiens de sortie du PF. En raison de l'évaluation incomplète des patientes dans tous les établissements, nous avons effectué des analyses distinctes de la qualité des soins de PF par établissement et par patient, par pays, plutôt qu'une seule analyse globale utilisant une variable dépendante représentant la qualité des soins de PF. Comme indiqué, les agents de collecte des données de SPA ne revenaient pas dans des établissements où aucune patiente n'était venue pour bénéficier des services proposés le jour de la visite. Par conséquent, notre échantillon peut représenter des établissements plus fréquentés qui disposent de moins de temps pour les patientes et par conséquent moins performants pour les indicateurs au niveau des patientes que des établissements moins fréquentés et disposant de plus de temps pour fournir un service de meilleure qualité aux patientes du PF. Quatrièmement, notre dichotomisation basée sur les données de qualité des soins de PF peut limiter la comparaison de nos résultats par rapport à d'autres études. Toutefois, le schéma de dichotomisation donne des indications sur la performance des établissements intégrés par rapport aux établissements non intégrés dans les pays, en fonction des niveaux de base de qualité des soins de PF

fournis dans chaque pays. Enfin, notre analyse s'est concentrée sur les établissements de soins de santé primaires et secondaires, car presque tous les établissements de soins tertiaires ont satisfait aux critères d'intégration. Les résultats ne doivent pas être interprétés pour les établissements de soins tertiaires dans ces pays.

Notre étude avait de nombreux atouts. Nous avons utilisé les données les plus récentes et les plus représentatives disponibles au niveau national, nous offrant ainsi des informations précieuses pouvant éclairer l'élaboration des politiques en cours concernant les programmes intégrés de PF. La présente étude est l'une des rares à s'appuyer sur les données de SPA, évaluation qui est relativement sous-utilisée compte tenu du besoin critique de recherche sur les thèmes du renforcement des systèmes de santé dans les pays en développement. Nous avons utilisé le processus de QIQ pour effectuer une évaluation théorique de la qualité des soins de PF, qui ne repose pas sur des mesures subjectives uniques de la qualité, comme cela est courant dans la littérature existante. En conséquence, notre évaluation de la qualité des soins de PF en milieu intégré et non intégré constitue une contribution importante à la base de données probantes.

Conclusion

Des recherches sur le lien entre l'intégration des services de VIH et la qualité des soins de PF sont nécessaires pour s'assurer que l'intégration des services se traduise par des soins de haute qualité qui améliorent la prestation des services et profitent à la santé des patientes. À l'aide de données sur la prestation de services au Malawi et en Tanzanie, nous avons constaté que l'intégration a un impact positif sur la qualité des soins de PF. Cependant, les résultats ont été mitigés au niveau des patientes.

Nos résultats n'ont pas confirmé les inquiétudes concernant les conséquences négatives potentielles de l'intégration des services VIH et PF. Bien que les recherches sur les points de vue des parties prenantes concernant la mise en œuvre de l'intégration indiquent que l'intégration peut surcharger l'emploi du

temps des établissements et nuire à la qualité des soins [4], nous avons relevé une seule association négative indiquant que l'intégration des services liés au VIH pouvait avoir une incidence négative sur la pratique des prestataires (c'est-à-dire une probabilité réduite de temps d'attente acceptable en Tanzanie). D'une manière générale, nos résultats suggèrent que la qualité des soins de PF peut être équivalente ou supérieure dans les établissements intégrés par rapport aux établissements non intégrés au Malawi et en Tanzanie. Des recherches supplémentaires sont nécessaires pour comprendre comment l'intégration des services de lutte contre le VIH peut influer sur la qualité des soins de PF dans divers contextes et comment la plateforme sur laquelle le PF est intégré peut influencer différemment la qualité des soins de PF.

Abréviations

IC : intervalle de confiance

PF : planning familial

RC : rapports de cote

QIQ : Quick Investigation of Quality (enquête rapide sur la qualité)

SPA : Service Provision Assessment (évaluation des prestations de services)

Concernant ce supplément

Cet article a été publié comme partie de l'ouvrage *Reproductive Health*, Volume 16 Supplement 1, 2019: Effective Integration of Sexual Reproductive Health and HIV Prevention, Treatment, and Care Services across sub-Saharan Africa: Where is the evidence for program implementation?
Le supplément a été publié dans le cadre d'une collaboration entre *Reproductive Health* et *BMC Public Health.* L'intégralité du contenu, avec les versions en français, en portugais et en anglais, est disponible en ligne :
https://bmcpublichealth.biomedcentral.com/articles/supplements/volume-19-supplement-1
et
https://reproductive-health-journal.biomedcentral.com/articles/supplements/volume-16-supplement-1

Déclarations

Approbation éthique et accord de participation

L'Office of Human Research and Ethics de l'Université de Caroline du Nord, à Chapel Hill, a déterminé que cette analyse de données secondaires ne constituait pas un sujet de recherche humain, tel que défini par la réglementation fédérale, et ne nécessitait donc pas l'approbation d'un comité d'examen

institutionnel. Le consentement et l'approbation éthique ont été obtenus par ICF Macro (le Program DHS) avant la collecte des données.

Accord de publication
Non applicable, il n'y a pas de données personnellement identifiables au niveau individuel.

Disponibilité des données et supports
Les données d'évaluation de la prestation de services anonymisées sont disponibles pour un usage public sous réserve d'inscription sur la page http://dhsprogram.com.

Conflits d'intérêts
Les auteurs déclarent ne pas avoir de conflits d'intérêts.

Financement
Le supplément de la revue est rendu possible grâce au soutien généreux du peuple américain via la United States Agency for International Development (USAID) en partenariat avec le Fonds des Nations unies pour la population (FNUAP) et le Programme commun des Nations Unies sur le VIH/SIDA (ONUSIDA). L'assistance générale est fournie par le Carolina Population Center et sa subvention du NIH Center (P2C HD050924).

Les opinions exprimées dans la présente publication sont celles des auteurs et ne reflètent pas nécessairement les politiques officielles de l'USAID, du FNUAP ou de l'ONUSIDA, la mention des dénominations de ministères ou d'organismes n'implique pas non plus l'aval du gouvernement américain, du FNUAP ou de l'ONUSIDA.

Contributions des auteurs
JBO et CM ont conçu et conceptualisé l'étude ; MC a contrôlé et s'est assuré de la qualité des données ; MC, JBO, CM ont analysé les données ; et tous ont interprété de manière approfondie les données. CM et MC ont rédigé l'article ; et JBO l'a révisé de manière approfondie. Tous les auteurs ont lu et approuvé la version finale du manuscrit.

Références

1. Johnson K, Varallyay I, Ametepi P. Integration of HIV and family planning services in sub-Saharan Africa: A review of the literature, current recommendations, and evidence from the service provision assessment health faculty surveys [Internet]. Calverton, Maryland, USA: ICF International; 2012 Sep. Report No.: 30. [accessed 2017 Sep 7] Available from: http://www.dhsprogram.com/publications/publication-AS30-Analytical-Studies.cfm.

2. Barden-O'Fallon J, Adamou B, Mejia C, Agala CB. A Review of Family Planning Outcomes in Integrated Health Programs and Research Recommendations — MEASURE Evaluation [Internet]. University of North Carolina at Chapel Hill; 2017 Feb. [accessed 2017 Jul 10] Available from: https://www.measureevaluation.org/resources/publications/wp-17-176.

3. Foreit KGF, Hardee K, Agarwal K. When does it make sense to consider integrating STI and HIV services with family planning services? Int Fam Plan Perspect. 2002;105–7.

4. MEASURE Evaluation. Findings from a Multi-Country Assessment of Integrated Health Programs [Internet]. Chapel Hill, NC, USA: University of North Carolina at Chapel Hill; 2014 Aug. [accessed 2017 Sep 10] Available from: https://www.measureevaluation.org/resources/publications/tr-14-115.

5. Ringheim K, Yeakey M, Gribble J, Sines E, Stepahin S. Supporting the integration of family planning and HIV services. [Internet]. Washington, DC, USA: Population Reference Bureau; 2009 Sep. [accessed 2017 Sep 7] Available from: http://www.popline.org/node/557931.

6. Duerr A, Hurst S, Kourtis AP, Rutenberg N, Jamieson DJ. Integrating family planning and prevention of mother-to-child HIV transmission in resource-limited settings. The Lancet. 2005;366:261–3.

7. Church K, Mayhew SH. Integration of STI and HIV prevention, care, and treatment into family planning services: a review of the literature. Stud Fam Plann. 2009;40:171–86.

8. Stover J, Fuchs N, Halperin D, Gibbons A, Gillespie D. Costs and benefits of adding family planning to services to prevent mother-to-child transmission of HIV (PMTCT). How family planning can increase the benefits of PMTCT by saving lives and reducing the number of orphans. [Internet]. 2003 Jul. [accessed 2017 Sep 7] Available from: http://www.popline.org/node/276464.

9. Adamchak S, Janowitz B, Liku J, Munyambanza E, Grey T, Keyes E. Study of family planning and HIV integrated services in five countries [Internet]. Research Triangle Park, NC, USA: Family Health International; 2010. [accessed 2017 Sep 8] Available from: http://addiscontinental.edu.et/files/FPHIVInt5countryreport.pdf.

10. Sherr L. Literature review on program strategies and models of continuity of HIV/maternal newborn and child health care for HIV-positive mothers and their HIV-positive/-exposed children. [Internet]. Arington, VA, USA: AIDS Support and Technical Assistance Resources, Task Order 1 (AIDSTAR-One); 2012 Feb. [accessed 2017 Sep 8] Available from: http://www.popline.org/node/561593.

11. Bruce J. Fundamental Elements of the Quality of Care: A Simple Framework. Stud Fam Plann. 1990;21:61.

12. Spaulding AB, Brickley DB, Kennedy C, Almers L, Packel L, Mirjahangir J, et al. Linking family planning with HIV/AIDS interventions: a systematic review of the evidence: AIDS. 2009;23:S79–88.

13. Coyne KM, Hawkins F, Desmond N. Sexual and reproductive health in HIV-positive women: a dedicated clinic improves service. Int. J. STD AIDS. 2007;18:420–1.

14. PATH. Preventing mother-to-child HIV transmission [Internet]. PATH; 2006 Aug. [accessed 2017 Sep 7] Available from: https://www.path.org/publications/files/ER_directions_summer06.pdf.

15. Mullick S, Askew I, Maluka T, Khoza D, Menziwa M. Integrating counselling and testing into family planning services: What happens to the existing quality of family planning when HIV services are integrated in South Africa? Addis Ababa, Ethiopia; 2006. p. 9–10.

16. Reynolds HW, Liku J, Beaston-Blaakman A, Kimani J, Burke H. Integrating family planning services into voluntary counseling and testing centers in Kenya. Operations research results. [Internet]. Research Triangle Park, NC, USA: Family Health International; 2006 Jul. [accessed 2017 Sep 7] Available from: http://www.popline.org/node/179189.

17. Ministry of Health- MoH/Malawi, ICF International. Malawi Service Provision Assessment 2013-14 [Internet]. Lilongwe, Malawi, and Rockville, MD, USA: MoH/Malawi and ICF International; 2014. [accessed 2017 Sep 8] Available from: http://dhsprogram.com/publications/publication-spa20-spa-final-reports.cfm.

18. Ministry of Health and Social Welfare/Tanzania, Ministry of Health/Zanzibar, National Bureau of Statistics/Tanzania, Office of Chief Government Statistician/Tanzania, ICF International. Tanzania Service Provision Assessment Survey 2014-2015 [Internet]. Dar es Salaam, Tanzania and Rockville, MD, USA: MoHSW, MoH, NBS, OCGS, and ICF International; 2016 Feb. [accessed 2017 Sep 8] Available from: http://www.dhsprogram.com/publications/publication-spa22-spa-final-reports.cfm

19. United States Agency for International Development (USAID). The DHS Program - Service Provision Assessments (SPA) [Internet]. [cited 2017 Jul 10]. [accessed 2017 Jul 10] Available from: http://dhsprogram.com/What-We-Do/Survey-Types/SPA.cfm.

20. MEASURE Evaluation. Quick Investigation of Quality: A User's Guide for Monitoring Quality of Care in Family Planning (2nd ed.) [Internet]. Chapel Hill, NC, USA: MEASURE Evaluation, University of North Carolina; 2016 Jan. [accessed 2017 Jul 10] Available from: https://www.measureevaluation.org/resources/publications/ms-15-104.

21. Windisch R, Waiswa P, Neuhann F, Scheibe F, de Savigny D. Scaling up antiretroviral therapy in Uganda: using supply chain management to appraise health systems strengthening. Glob Health. 2011;7:25.

22. El-Sadr WM, Holmes CB, Mugyenyi P, Thirumurthy H, Ellerbrock T, Ferris R, et al. Scale-up of HIV Treatment Through PEPFAR: A Historic Public Health Achievement. J Acquir Immune Defic Syndr 1999. 2012;60:S96-104.

23. Schouten EJ, Jahn A, Ben-Smith A, Makombe SD, Harries AD, Aboagye-Nyame F, et al. Antiretroviral drug supply challenges in the era of scaling up ART in Malawi. J Int AIDS Soc 2011;14:S4.

Tableaux

Tableau 1 Liste des Indicateurs de QIQ

Numéro d'Indicateur QIQ	Description d'Indicateur QIQ
1	Le prestataire démontre de bonnes compétences en matière de conseil[a]
1a	Consultation et annotation du dossier de patiente
1b	Utilisation d'aides visuelles
1c	Confidentialité visuelle et auditive assurée
2	Le prestataire assure la patiente du respect de la vie privée
3	Le prestataire interroge la patiente sur ses intentions en matière de procréation
4	Le prestataire discute avec la patiente de la méthode qu'elle préférerait
5	Le prestataire aborde le sujet du VIH/SIDA (lance le sujet ou apporte des réponses)
6	Le prestataire discute de l'utilisation de la méthode double
7	Le prestataire traite la patiente avec respect et dignité[b]
8	Le prestataire adapte les informations clés aux besoins particuliers de la patiente
9	Le prestataire donne des informations précises sur la méthode acceptée (par exemple, le mode d'emploi, les effets secondaires et les complications)
10	Le prestataire donne des instructions sur la prochaine visite
11	Le prestataire suit les procédures de contrôle des infections décrites dans les directives
12	Le prestataire reconnaît/identifie les contre-indications conformes aux directives[b]
13	Le prestataire effectue les procédures cliniques conformément aux directives
14	Le personnel traite la patiente avec dignité et respect
15	La patiente participe activement à la discussion et au choix de la méthode (la patiente est « responsabilisée »)
16	La patiente reçoit la méthode qu'elle a choisie[b]
17	La patiente estime que le prestataire gardera ses informations confidentielles[b]
18	L'établissement dispose de toutes les méthodes (approuvées) disponibles ; pas de rupture de stock
19	L'établissement dispose des produits de base nécessaires à la fourniture des méthodes disponibles via ses services (équipement de stérilisation, gants, brassard de tensiomètre, spéculums, éclairage adéquat et eau)
20	L'établissement offre une intimité pour l'examen pelvien/l'insertion du DIU (personne ne peut voir)[c]
21	L'établissement dispose de mécanismes pour effectuer des modifications de programme en fonction des retours des patientes
22	L'établissement a reçu une visite de contrôle au cours des 6 derniers mois[d]
23	L'établissement dispose d'un lieu de stockage adéquat des contraceptifs et des médicaments (à l'abri de l'eau, de la chaleur, de la lumière directe du soleil) dans ses locaux
24	L'établissement dispose de directives cliniques de pointe
25	Le temps d'attente est acceptable

[a]Traité via trois sous-indicateurs dans l'analyse (1a-1c)
[b]Aucune correspondance de données disponible
[c]Exclus
[d]Nous fixons le seuil à 6 mois

Tableau 2 Caractéristiques des établissements non intégrés et intégrés (SPA du Malawi 2013–2014) (n = 305)

	Non intégré (n = 184)		Intégré (n = 121)		Total (n = 305)		*valeur p*
	n	%	n	%	n	%	
**Autorité de gestion**							0,155
Gouvernement/Secteur public	32	18,1	33	27,4	65	21,8	
Secteur privé (à but non lucratif)[a]	33	17,7	20	16,4	53	17,2	
Secteur privé (à but lucratif)[b]	119	64,2	68	56,1	187	61,0	
**Type d'établissement**							0,016
Dispensaire	20	10,4	21	17,5	41	13,2	
Clinique	148	79,9	97	80,0	245	80,0	
Poste de santé/Maternité[c]	16	9,7	3	2,5	19	6,8	
**Lieu**							0,156
Urbain	90	48,6	69	56,9	159	51,9	
Rural	94	51,4	52	43,1	146	48,2	
**Zone**							0,006
Nord	18	9,2	21	16,6	39	12,1	
Centre est	30	16,0	7	5,7	37	11,9	
Centre ouest	48	25,7	36	29,4	84	27,2	
Sud-est	29	16,1	28	23,9	57	19,2	
Sud-ouest	59	33,0	29	24,4	88	29,7	

[a]Le secteur privé (à but non lucratif) est composé des établissements qui sont déclarés comme « Christian Health Association of Malawi (CHAM) », « Organisme/mission religieux/se (non-CHAM) » ou « Organisation Non Gouvernementale ».

[b]Le secteur privé (à but lucratif) comprend les établissements ayant déclaré une autorité de gestion « Privée (à but lucratif) » ou « Société ». [c]En raison du faible nombre de maternités dans l'échantillon analytique, les termes « Maternité » et « Poste de santé » ont été regroupés sous un seul type d'établissement global.

Tableau 3 Caractéristiques des établissements non intégrés et intégrés (SPA de Tanzanie 2014–2015) (n = 750)

	Non intégré (n = 354)		Intégré (n = 396)		Total (n = 750)		*valeur p*
	n	%	n	%	n	%	
**Autorité de gestion**							0,260
Gouvernement/Secteur public	284	86,4	360	90,3	644	87,9	
Hors gouvernement/Secteur public[a]	70	13,6	36	9,7	106	12,1	
**Type d'établissement**							<0,0001
Centre de santé/Clinique[b]	82	5,0	263	24,1	345	12,2	
Dispensaire	272	95,0	133	76,0	405	87,8	
**Lieu[c]**							0,543
Urbain	70	15,7	80	18,0	150	16,6	
Rural	284	84,3	316	82,0	600	83,4	

[a]Le Gouvernement/Secteur public comprend les établissements qui sont déclarés comme « Privés », « Organisme/mission religieux/se » ou « Autres (secteur parapublic et défense/prison/police) ».
[b]En raison du petit nombre de cliniques de l'échantillon analytique, « Clinique » et « Centre de santé » ont été regroupés sous un seul type d'installation global.
[c]Estimations régionales non présentées.

Tableau 4 Pourcentage des établissements non intégrés et intégrés et patientes répondant à chacun des critères des Indicateurs QIQ (SPA du Malawi 2013–2014)

N° Indicate	Description	Non intégré	Intégré	Total	*valeur p*
Inventaire au niveau de l'établissement (n = 305)		%	%	%	
11	Le prestataire (dans l'établissement) suit les procédures de contrôle des infections décrites dans les directives	60	63	61	0,620
18	L'établissement dispose de toutes les méthodes (approuvées) disponibles ; pas de rupture de stock	46	58	50	0,039
19	L'établissement dispose des produits de base nécessaires à la fourniture des méthodes disponibles via ses services (équipement de stérilisation, gants, brassard de tensiomètre, spéculums, éclairage adéquat et eau)	36	35	36	0,902

21	L'établissement dispose de mécanismes pour effectuer des modifications de programme en fonction des retours des patientes	25	30	27	0,370
22	L'établissement a reçu une visite de contrôle au cours des 6 derniers mois[a]	64	80	71	0,003
23	L'établissement dispose d'un lieu de stockage adéquat des contraceptifs et des médicaments (à l'abri de l'eau, de la chaleur, de la lumière directe du soleil) dans ses locaux	69	78	72	0,087
24	L'établissement dispose de directives cliniques de pointe	45	48	46	0,629
Observation PF au niveau des patientes (n = 323)					
1a	Consultation et annotation du dossier de patiente	84	96	89	0,005
1b	Utilisation d'aides visuelles	20	21	20	0,907
1c	Confidentialité visuelle et auditive assurée	90	82	87	0,334
2	Le prestataire assure la patiente du respect de la vie privée	32	22	28	0,266
3	Le prestataire interroge la patiente sur ses intentions en matière de procréation	29	26	28	0,688
5	Le prestataire aborde le sujet du VIH/SIDA (lance le sujet ou apporte des réponses)	8	15	11	0,207
6	Le prestataire discute de l'utilisation de la méthode double	6	15	10	0,095
9	Le prestataire donne des informations précises sur la méthode acceptée (par exemple, le mode d'emploi, les effets secondaires et les complications)[b]	57	63	60	0,552
10	Le prestataire donne des instructions sur la prochaine visite	84	94	88	0,050
13	Le prestataire effectue les procédures cliniques conformément aux directives[c]	57	60	58	0,795
15	La patiente participe activement à la discussion et au choix de la méthode (la patiente est « responsabilisée »)	42	47	44	0,633
Entretien de sortie du PF au niveau des patientes (n = 315)					
4	Le prestataire discute avec la patiente de la méthode qu'elle préférerait	35	32	34	0,635
8	Le prestataire adapte les informations clés aux besoins particuliers de la patiente	93	91	92	0,546
14	Le personnel traite la patiente avec dignité et respect[d]	99	98	99	0,236
25	Temps d'attente acceptable	87	85	86	0,732

[a] Pour cette étude, le seuil a été fixé à 6 mois.

[b] Neuf cas n'ont pas obtenu de méthode et ont donc été exclus.

[c] Quarante-six cas n'ont pas fait l'objet d'une procédure clinique et ont donc été exclus.

[d] Un cas n'a pas fourni d'information pour cet indicateur et a donc été exclu.

Tableau 5 Pourcentage des établissements non intégrés et intégrés répondant à chacun des critères des Indicateurs QIQ (SPA de Tanzanie 2014–2015)

N° Indicateur	Description	Non intégré	Intégré	Total	*valeur p*
Inventaire au niveau de l'établissement (n = 750)		%	%	%	
11	Le prestataire (dans l'établissement) suit les procédures de contrôle des infections décrites dans les directives	61	62	62	0,921
18	L'établissement dispose de toutes les méthodes (approuvées) disponibles ; pas de rupture de stock	54	68	59	0,003
19	L'établissement dispose des produits de base nécessaires à la fourniture des méthodes disponibles via ses services (équipement de stérilisation, gants, brassard de tensiomètre, spéculums, éclairage adéquat et eau)	11	15	13	0,232
21	L'établissement dispose de mécanismes pour effectuer des modifications de programme en fonction des retours des patientes	24	34	27	0,019
22	L'établissement a reçu une visite de contrôle au cours des 6 derniers mois	90	95	92	0,104
23	L'établissement dispose d'un lieu de stockage adéquat des contraceptifs et des médicaments (à l'abri de l'eau, de la chaleur, de la lumière directe du soleil) dans ses locaux	42	57	47	0,002
24	L'établissement dispose de directives cliniques de pointe	56	63	58	0,132
Observation PF au niveau des patientes (n = 1060)					
1a	Consultation et annotation du dossier de patiente	72	78	75	0,236
1b	Utilisation d'aides visuelles	12	14	13	0,439
1c	Confidentialité visuelle et auditive assurée	75	72	74	0,662
2	Le prestataire assure la patiente du respect de la vie privée	41	34	37	0,235
3	Le prestataire interroge la patiente sur ses intentions en matière de procréation	34	33	33	0,867
5	Le prestataire aborde le sujet du VIH/SIDA (lance le sujet ou apporte des réponses)	19	22	21	0,530
6	Le prestataire discute de l'utilisation de la méthode double	6	8	7	0,414
9	Le prestataire donne des informations précises sur la méthode acceptée (par exemple, le mode d'emploi, les effets secondaires et les complications)[a]	64	61	63	0,643
10	Le prestataire donne des instructions sur la prochaine visite	83	82	83	0,829

13	Le prestataire effectue les procédures cliniques conformément aux directives[b]	54	53	54	0,883
15	La patiente participe activement à la discussion et au choix de la méthode (la patiente est « responsabilisée »)	50	51	50	0,942
Entretien de sortie du PF au niveau des patientes (n = 1059)					
4	Le prestataire discute avec la patiente de la méthode qu'elle préférerait	33	37	35	0,359
8	Le prestataire adapte les informations clés aux besoins particuliers de la patiente	97	95	96	0,628
14	Le personnel traite la patiente avec dignité et respect	97	94	95	0,106
25	Temps d'attente acceptable	84	71	77	0,005

[a]Trente-sept cas n'ont pas obtenu de méthode et ont donc été exclus.
[b]Un total de 291 cas n'a pas subi de procédure clinique. Un cas n'a rapporté aucune information pour l'indicateur. Tous ont été exclus.

Tableau 6 Association du statut d'intégration avec la qualité des soins de PF (SPA du Malawi 2013–2014)

	Au niveau de l'établissement				Au niveau des patientes[a]	
	Non ajusté		Ajusté		Non ajusté	
	OU	(IC 95 %)	OU	(IC 95 %)	OU	(IC 95 %)
Services de VIH Intégrés	2,18[b]	(1,36, 3,50)	2,24[b]	(1,32, 3,79)	1,05	(0,48, 2,31)
Autorité de gestion						
Gouvernement/Secteur public			réf.			
Secteur privé (à but non lucratif)			5,42[b]	(1,64, 17,91)		
Secteur privé (à but lucratif)			0,64	(0,23, 1,77)		
Type d'établissement						
Dispensaire			réf.			
Clinique			2,21	(0,67, 7,26)		
Poste de santé/Maternité			0,23[c]	(0,05, 0,97)		
Lieu						
Urbain			réf.			
Rural			1,32	(0,73, 2,40)		
Zone						
Nord			réf.			
Centre est			0,47	(0,16, 1,38)		
Centre ouest			1,06	(0,43, 2,59)		
Sud-est			0,63	(0,25, 1,64)		
Sud-ouest			1,50	(0,61, 3,69)		
N	305		305		323	

[a]Les Indicateurs 9 et 13 sont exclus en raison du nombre moins élevé de réponses de services non applicables pour certains répondants.

[b]$p < 0{,}01$

[c]$p < 0{,}05$

Les analyses au niveau des patientes tiennent compte du regroupement des patientes dans des établissements.

Tableau 7 Association du statut d'intégration avec la qualité des soins de PF (SPA de Tanzanie 2014–2015)

	Au niveau de l'établissement				Au niveau des patientes[a]	
	Non ajusté		Ajusté		Non ajusté	
	OU	(IC 95 %)	OU	(IC 95 %)	OU	(IC 95 %)
Services de VIH Intégrés	2,26[b]	(1,51, 3,37)	2,06[c]	(1,37, 3,22)	0,91	(0,55, 1,51)
Autorité de gestion						
Gouvernement/Secteur public			réf.			
Hors Gouvernement/Secteur public			0,73	(0,35, 1,53)		
Type d'établissement						
Centre de santé/Clinique			réf.			
Dispensaire			0,52[d]	(0,33, 0,83)		
Lieu						
Urbain			réf.			
Rural			0,62	(0,33, 1,14)		
N	750		750		1060	

[a] Les Indicateurs 9 et 13 sont exclus en raison du nombre moins élevé de réponses de services non applicables pour certains répondants.

[b] $p < 0,001$
[c] $p < 0,05$
[d] $p < 0,01$

Les analyses au niveau des patientes tiennent compte du regroupement des patientes dans des établissements.
Les estimations régionales sont maintenant affichées.

Fichier additionnel 1

Tableau S1 Description des mesures QIQ et SPA mises en correspondance dans la présente étude

QIQ		SPA			
N° Indicateur QIQ	Description d'Indicateur QIQ	Instrument SPA	Description des mesures SPA mises en correspondance		Description des indicateurs QIQ dichotomiques créés pour les analyses
1	Le prestataire démontre de bonnes compétences en matière de conseil	Observation	I1A	Dossier de patiente consulté et annoté	1 = dossier de patiente consulté et annoté, 0 = dossier de patiente non consulté et non annoté.
			I1B	Utilisation d'aides visuelles	1 = utilisation d'aides visuelles, 0 = non-utilisation d'aides visuelles.
			I1C	Confidentialité visuelle et auditive assurée	1 = confidentialité visuelle et auditive assurée, 0 = confidentialité visuelle et auditive non assurée.
2	Le prestataire assure la patiente du respect de la vie privée	Observation	A assuré la patiente oralement du respect de la confidentialité		1 = confidentialité assurée oralement, 0 = confidentialité non assurée oralement.
3	Le prestataire interroge la patiente sur ses intentions en matière de procréation	Observation	Désir d'un enfant ou de plusieurs enfants		1 = désir d'un enfant OU programmation des naissances demandée, 0 = aucune demande.
			Moment souhaité pour la naissance du prochain enfant		
4	Le prestataire discute avec la patiente de la méthode qu'elle préférerait	Entretien avec la patiente	[Un ensemble de mesures demandant si la patiente avait pensé à la méthode du PF avant la visite.]		1 = le prestataire a parlé à la patiente de la méthode d'intérêt<br>0 = le prestataire n'a pas parlé à la patiente de la méthode d'intérêt<br>0 = OU la patiente n'a pas pensé à changer de méthode<br>0 = OU la patiente n'a pas pensé à la méthode qu'elle souhaitait utiliser<br>0 = OU la patiente a cessé d'utiliser la méthode (par choix - pas de problèmes).
5	Le prestataire aborde le sujet du VIH/SIDA (lance le sujet ou apporte des réponses)	Observation	Risque perçu d'IST/du VIH par la patiente		1 = discussion sur le risque d'IST/du VIH perçu par la patiente OU sur l'utilisation de préservatifs, 0 = aucune discussion sur le sujet.
			Utilisation de préservatifs pour prévenir les IST/le VIH		
6	Le prestataire discute	Observation	Utilisation de		1 = discussion de l'utilisation

	de l'utilisation de la méthode double		préservatifs avec une autre méthode (méthode double) pour optimiser la protection	d'une méthode double, 0 = aucune discussion sur le sujet.
7	Le prestataire traite la patiente avec respect et dignité	Aucune correspondance de données disponible.		
8	Le prestataire adapte les informations clés aux besoins particuliers de la patiente	Entretien avec la patiente	Quantité d'explications que vous avez reçues sur le problème ou le traitement	1 = aucun problème et aucune explication reçue, 0 = problème mineur, problème majeur ou ne sait pas.
9	Le prestataire donne des informations précises sur la méthode acceptée (mode d'emploi, effets secondaires, complications)	Observation	[Un ensemble de tâches détaillées d'informations à communiquer par méthode.]	1 = valeur égale ou supérieure à la médiane des informations requises communiquées pour au moins une procédure sélectionnée fournie ou prescrite, 0 = valeur inférieure à la médiane des informations requises communiquées pour toutes les procédures sélectionnées.
10	Le prestataire donne des instructions sur la prochaine visite	Observation	Discussion à ce sujet lors d'une prochaine visite	1 = discussion lors d'une prochaine visite, 0 = pas de discussion.
11	Le prestataire suit les procédures de contrôle des infections décrites dans les directives	Inventaire	[Un ensemble de précautions et de conditions standard pour l'examen de la patiente (par exemple, eau courante, savon pour le lavage des mains).]	1 = nombre égal ou supérieur au nombre médian de mesures de précaution de prévention des infections observées, 0 = en deçà du nombre médian de mesures.
12	Le prestataire reconnaît/identifie les contre-indications conformes aux directives	Aucune correspondance de données disponible.		
13	Le prestataire effectue les procédures cliniques conformément aux directives	Observation	[Un ensemble de tâches cliniques détaillées à exécuter par procédure clinique.]	1 = valeur égale ou supérieure à la médiane dans les tâches requises avant/pendant/après la procédure pour au moins une procédure, 0 = valeur en deçà de la médiane pour

				toutes les procédures sélectionnées.
14	Le personnel traite la patiente avec dignité et respect	Entretien avec la patiente	Comment le personnel vous a traitée	1 = aucun problème avec la façon dont le personnel vous a traitée, 0 = problème mineur, problème majeur ou ne sait pas.
15	La patiente participe activement à la discussion et au choix de la méthode (la patiente est « responsabilisée »)	Observation	La patiente a exprimé des préoccupations au sujet de la méthode ou a posé des questions sur la méthode, notamment sur les effets secondaires potentiels de la méthode.	1 = la patiente a exprimé des préoccupations […], notamment sur les effets secondaires potentiels de la méthode, 0 = n'a exprimé aucune préoccupation.
16	La patiente reçoit la méthode qu'elle a choisie	Aucune correspondance de données disponible.		
17	La patiente estime que le prestataire gardera ses informations confidentielles	Aucune correspondance de données disponible.		
18	L'établissement dispose de toutes les méthodes (approuvées) disponibles ; pas de rupture de stock	Inventaire	[Un ensemble de méthodes fournies et en stock (c'est-à-dire au moins un produit contraceptif valide observé par méthode).]	1 = au moins 4 méthodes fournies et en stock (au moins une valide), 0 = autre.
19	L'établissement dispose des produits de base nécessaires à la fourniture des méthodes disponibles via ses services (équipement de stérilisation, gants, brassard de tensiomètre, spéculums, éclairage adéquat et eau)	Inventaire	[Un ensemble de l'équipement de base et des fournitures observés dans les établissements.]	Non destinés aux DIU : 1 = tensiomètre (numérique ou manuel) et lampe d'examen observés, 0 = autre.<br>Destinés aux DIU : 1 = tensiomètre (numérique ou manuel), lampe d'examen et spéculum (toute taille) observés, 0 = autre.
20	L'établissement offre une intimité pour l'examen pelvien/l'insertion du DIU (personne ne peut voir)	Exclus.		
21	L'établissement dispose	Inventaire	[Un ensemble de	1 = oui, procédure d'examen

	de mécanismes pour effectuer des modifications de programme en fonction des retours des patientes		mesures relatives à la mise en œuvre d'un système permettant de déterminer les retours des patientes et l'existence d'une procédure permettant d'analyser les commentaires de retours.]	ou de compte rendu de l'opinion de la patiente<br>0 = pas de procédure pour examiner ou rendre compte de l'opinion de la patiente<br>0 = OU pas de système pour déterminer l'opinion des patientes<br>0= OU ne sait pas s'il existe une procédure pour examiner ou rendre compte de l'opinion des patientes<br>0 = OU oui, il existe un système permettant de connaître l'opinion des patientes, mais ne sait pas s'il existe une procédure permettant d'examiner ou de rendre compte de l'opinion des patientes
22	L'établissement a reçu une visite de contrôle au cours des __ derniers mois	Inventaire	De quand date la dernière visite d'un contrôleur extérieur à l'établissement pour une visite de contrôle ?	1 = visite dans les 6 mois, 0 = visite il y a plus de 6 mois<br>0 = OU pas de contrôle externe.

QIQ : Quick Investigation of Quality (enquête rapide sur la qualité)

SPA : Service Provision Assessment (évaluation des prestations de services)
