# Supplementary material for: Quality of family planning services in HIV integrated and non-integrated health facilities in Malawi and Tanzania
Source: Reprod Health. 2019 May 29;16(Suppl 1):58. doi: 10.1186/s12978-019-0712-y (PMC6538555; doi:10.1186/s12978-019-0712-y)
Supplement: Supplementary file 3 — Translation of the abstract of this article into Portuguese. (PDF 115 kb) [file 12978_2019_712_MOESM3_ESM.pdf]

Reproductive Health 2019, 16 (Suppl 1) [translated from the original publication]

A qualidade dos serviços de planeamento familiar em instalações de saúde integradas e não integradas de VIH no Maláui e na Tanzânia

Michael A. Close[1] , Janine Barden-O'Fallon[1*] , Carolina Mejia[1]

[1]Carolina Population Center, Health Behavior Department, Gillings School of Global Public Health,
University of North Carolina at Chapel Hill, Chapel Hill, NC, USA

MC: mclose@email.unc.edu
JB: bardenof@email.unc.edu
CM cmejia@unc.edu

*Autor correspondente: Janine Barden-O'Fallon

Resumo

Introdução:

A integração dos serviços de planeamento familiar (PF) e dos serviços relacionados com o VIH é comum na África Subsariana. Uma investigação residual analisou a forma como a qualidade dos cuidados de PF diverge entre as instalações integradas e não integradas. Através de dados representativos a nível nacional, analisou-se o modo como a integração do VIH está associada à qualidade dos cuidados de PF.

Métodos:

Os dados foram obtidos a partir do inquérito Service Provision Assessments (SPA) no Maláui (2013 – 2014) e na Tanzânia (2014 – 2015). A amostra analítica limitava-se às instalações de nível inferior no Maláui (n=305) e na Tanzânia (n=750) que ofereciam serviços de PF. Correspondemos as medidas do SPA a indicadores de qualidade dos cuidados de PF no Quick Investigation of Quality (QIQ). Foram realizadas análises bivariadas e multivariadas de 22 indicadores QIQ para averiguar como o estado da integração está relacionado com os indicadores QIQ individuais e a qualidade dos cuidados de PF por instalações e clientes.

Resultados:

A prevalência da integração do VIH no Maláui (39%) e na Tanzânia (38%) foi semelhante. A integração dos serviços de VIH está significativamente associada (p < 0,05) aos indicadores QIQ no Maláui (n=3) e na Tanzânia (n=4). Com exceção de uma associação negativa na Tanzânia, todas as outras associações foram positivas. Ao nível das instalações, a integração do VIH está associada ao aumento da probabilidade da qualidade dos cuidados de PF estar dentro, ou acima, da média no Maláui (OR ajustada = 2,24; intervalo de confiança (IC) de 95% = 1,32, 3,79) e na Tanzânia (OR ajustada = 2,10; IC de 95% = 1,37, 3,22). Ao nível dos clientes, a integração do VIH não está associada à qualidade dos cuidados de PF em nenhum dos países.

Conclusão:

A integração do VIH está aparentemente associada de forma vantajosa à qualidade dos cuidados de PF com base nas amostras do Maláui e da Tanzânia. Ao utilizar um espetro de indicadores da qualidade dos cuidados de PF, verificámos que existem poucos indícios para apoiar as dúvidas de que a integração do VIH poderá sobrecarregar as instalações e os fornecedores, bem como causar um impacto negativo nos resultados de qualidade. Em vez disso, parece reforçar a prestação dos serviços de PF, aumentado a probabilidade dos produtos armazenados de PF e alcançando outros indicadores de qualidade ao nível das instalações, eventualmente através de cadeias de abastecimento relacionadas com o VIH. É necessário realizar uma investigação mais aprofundada para avaliar os resultados da qualidade dos cuidados de PF através das várias plataformas de integração de PF localizadas na África Subsariana.

Palavras-chave: Qualidade do serviço, Índice de qualidade, Integração, Planeamento familiar, VIH, SPA, QIQ, Maláui, Tanzânia

Sobre este suplemento

Este artigo foi publicado como parte da revista científica *Reproductive Health*, Volume 16, Suplemento 1, 2019: Integração Eficaz dos Serviços de Saúde Sexual e Reprodutiva e de Prevenção, Cuidados e Tratamento do VIH na África Subsariana: Onde estão as provas da implementação do programa?
O suplemento foi publicado como uma colaboração entre as revistas científicas *Reproductive Health* e *BMC Public Health.* O conteúdo integral, incluindo as versões em francês, português e inglês, estão disponíveis online:
https://bmcpublichealth.biomedcentral.com/articles/supplements/volume-19-supplement-1
e
https://reproductive-health-journal.biomedcentral.com/articles/supplements/volume-16-supplement-1
